# Unfractionated Heparin Enhances Sepsis Prognosis Through Inhibiting Drp1‐Mediated Mitochondrial Quality Imbalance

**DOI:** 10.1002/advs.202407705

**Published:** 2024-10-24

**Authors:** Ruixue Liu, He Huang, Dongyao Hou, Shuai Hao, Qiao Guo, Haitang Liao, Rui Song, Yu Tian, Qian Chen, Zhenchun Luo, Daqing Ma, Liangming Liu, Chenyang Duan

**Affiliations:** ^1^ Department of Anesthesiology The Second Affiliated Hospital of Chongqing Medical University No. 76, Linjiang Road, Yuzhong District Chongqing 400010 P. R. China; ^2^ Department of Anesthesiology Taihe Hospital Hubei University of Medicine Shiyan 442099 P. R. China; ^3^ Research Institute of General Surgery, Jinling Hospital Affiliated Hospital of Medical School Nanjing University Nanjing 210002 P. R. China; ^4^ Department of Intensive Care Unit Chongqing Hospital of Traditional Chinese Medicine Chongqing 400013 P. R. China; ^5^ Perioperative and Systems Medicine Laboratory, Department of Anesthesiology National Clinical Research Center for Child Health, Children's Hospital Zhejiang University School of Medicine Hangzhou 310053 P. R. China; ^6^ Division of Anaesthetics, Pain Medicine and Intensive Care Department of Surgery and Cancer Faculty of Medicine Imperial College London, Chelsea & Westminster Hospital London SW10 9NH UK; ^7^ State Key Laboratory of Trauma and Chemical Poisoning Department of Shock and Transfusion, Daping Hospital Army Medical University Chongqing 400042 P. R. China

**Keywords:** Drp1, endothelial dysfunction, mitochondria quality, sepsis, unfractionated heparin

## Abstract

Unfractionated heparin (UFH) is commonly used as an anticoagulant in sepsis treatment and has recently been found to have non‐anticoagulant effects, but underlying mechanisms remain unclear. This retrospective clinical data showed that UFH has significant protective effects in sepsis compared to low‐molecular‐weight heparin and enoxaparin, indicating potential benefits of its non‐anticoagulant properties. Recombinant protein chip screening, surface plasmon resonance, and molecular docking data demonstrated that UFH specifically bound to the cytoplasmic Drp1 protein through its zone 2 non‐anticoagulant segment. In‐vitro experiments verified that UFH's specific binding to Drp1 suppressed Drp1 translocation to mitochondria following “sepsis” challenge, thereby improving mitochondrial morphology, function and metabolism in vascular endothelial cells. Consequently, UHF comprehensively protected mitochondrial quality, thus reducing vascular leakage and improving prognosis in a sepsis rat model. These findings highlight the potential of UFH as a sepsis treatment strategy targeting non‐anticoagulation mechanism.

## Graphical abstract

This study demonstrates that heparin (UFH) treats sepsis by non‐anticoagulant mechanisms, targeting Drp1 to enhance mitochondrial quality and reduce vascular leakage. Through clinical database analysis and experimental validation, UFH's Zone 2 specifically binds Drp1, inhibiting its mitochondrial activity and stabilizing endothelial function, providing a novel target for future heparin derivatives in sepsis management and critical care.

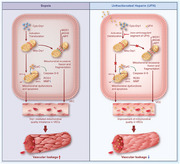

## 1 Introduction

Sepsis is a severe condition arising from infection‐induced organ dysfunction, representing a major cause of mortality in intensive care units (ICUs). It is characterized by a so‐called “storm” of inflammatory cytokines, endothelial damage, vascular leakage, and microthrombus formation, ultimately leading to inadequate tissue perfusion, organ failure, and death.^[^ ^1^ ^]^ The World Health Organization estimates that sepsis affects ≈30–50 million people globally each year,^[^ ^2^ ^]^ resulting in ≈10–15 million deaths, constituting nearly one‐fifth death globally.^[^ ^1^, ^3^ ^]^ This high prevalence and poor prognosis underscore the importance of sepsis as a global public health challenge. Although sepsis‐related mortality rate has been decreased in high‐income countries, they continue to be significantly higher in low‐ and middle‐income countries.^[^ ^3^ ^]^

Over the past few decades, several important sepsis treatment strategies have been introduced, such as activated protein C and early goal‐directed therapy (EGDT). However, substantial clinical evidence demonstrated their ineffectiveness in significantly reducing the mortality of patients with sepsis and removed from guidelines.^[^ ^4^ ^]^ For example, treatment with activated protein C has been associated with complications such as intracranial hemorrhage in patients with sepsis, which increases the risk of death.^[^ ^5^ ^]^ The data of three major clinical trials (ARISE, ProCESS, and ProMISe) revealed that the EGDT did not significantly reduce the mortality rate in patients with sepsis, and instead increased the requirement for resuscitation fluids, vasopressors, and dobutamine.^[^ ^6^ ^]^ Additionally, no significant differences were found in the outcomes of patients who received liberal and restrictive fluid therapy strategies for sepsis‐induced hypotension,^[^ ^7^ ^]^ indicating that current fluid management strategies require further research. The current sepsis guidelines primarily recommend early antibiotic treatment and volume resuscitation. However, the non‐specific nature of antibiotics, which relies heavily on the patient's immune response, leads to unpredictable results and challenges in standardization. Furthermore, an increase in antibiotic resistance complicates treatment and has become a significant risk factor for poor sepsis outcomes.^[^ ^8^ ^]^ Therefore, there is an urgent need to develop new sepsis treatment strategies, particularly those that target intracellular damage and focus on organ protection, to improve patient outcomes.

The prevalence of disseminated intravascular coagulation reaches up to 35% among patients with sepsis,^[^ ^9^ ^]^ highlighting the critical need for effective management strategies to tackle this complication. Accordingly, a key component of sepsis treatment involves the use of appropriate anticoagulant therapy to prevent the formation of microthrombi, thereby helping to control progression of the condition. Unfractionated heparin (UFH) is a widely used anticoagulant, which primarily binds to antithrombin III (AT III) to disrupt the coagulation cascade. Recent study indicates that the benefits of UFH use may be beyond its anticoagulation but include anti‐inflammatory, antiproliferative, and antiviral effects.^[^ ^10^ ^]^

Mounting evidence emphasizes the crucial role of mitochondrial quality imbalances in sepsis,^[^ ^11^ ^]^ especially in severe forms such as septic shock, as a critical determinant of disease progression, directly impacting the risk of multi‐organ failure and mortality.^[^ ^12^ ^]^ Consequently, maintaining or restoring mitochondrial quality has emerged as an important therapeutic strategy in sepsis management aimed at protecting multiple organs and improving patient outcomes. We recently uncovered a pivotal role of dynamin‐related protein 1 (Drp1) in the disruption of mitochondrial quality during sepsis.^[^ ^13^ ^]^ Drp1 is fundamentally involved in regulating mitochondrial fission and apoptosis, with notable alteration in these activities in pathological states, including sepsis.^[^ ^14^ ^]^ Specifically, activated Drp1 interacts with the mitochondrial protein BAX, facilitating its translocation to mitochondria and consequently triggering cell apoptosis.^[^ ^15^ ^]^ Therefore, this study further focused on the potential role of Drp1 in regulating mitochondrial dynamics to explore its therapeutics in sepsis beyond anticoagulation through various approaches including Drp1 recombinant protein chips, surface plasmon resonance (SPR) affinity detection, and molecular docking from cellular to animal model settings together with clinical database analysis.

## 2 Results

### 2.1 UFH Exerts a Significant Protective Effect on the Clinical Prognosis of Patients with Sepsis via a Non‐Anticoagulant Mechanism

**Figure 1:**
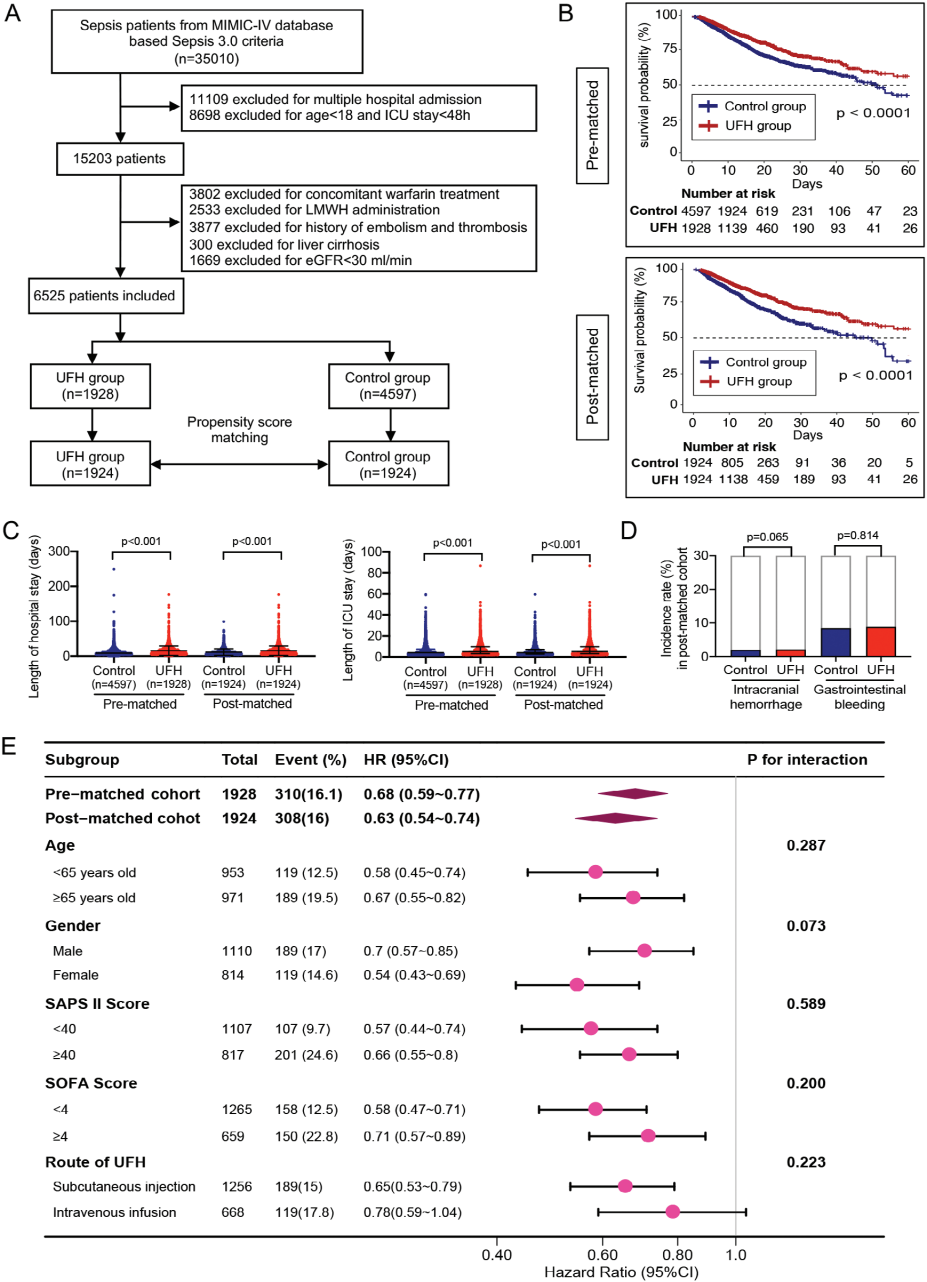
Efficacy of UFH therapy in sepsis outcomes. A) Flowchart illustrating the selection of patients with sepsis from the MIMIC‐IV database. B) Kaplan–Meier survival curves comparing survival rates of patients with sepsis with and without UFH therapy before (upper) and after (lower) propensity score matching. C) Duration of hospital and ICU stay before and after propensity score matching. D) Incidence of intracranial hemorrhage and gastrointestinal bleeding in patients following propensity score matching. E) Forest plot of hazard ratios (HRs) for mortality in pre‐ and post‐matched cohorts, analyzed according to age, sex, Simplified Acute Physiology Score (SAPS) II, Sequential Organ Failure Assessment (SOFA) score, and route of UFH administration. *P* < 0.05 was considered to indicate a statistically significant effect. LMWH, low‐molecular‐weight heparin; eGFR, estimated glomerular filtration rate.

We first performed a retrospective analysis of a clinical cohort to investigate the contribution of UFH on the outcomes of critically ill patients with sepsis. A total of 35010 patients diagnosed with sepsis (according to Sepsis 3.0 criteria) were obtained from the MIMIC‐IV database (https://physionet.org/content/mimiciv/2.2/). After filtering according to inclusion criteria of this study, 6525 patients were included for initial analysis and further analysis after propensity score matching (**Figure** **1**A). The clinical characteristics of the study population are summarized in Table S1 (Supporting Information). The Kaplan–Meier curve showed that in‐hospital mortality of the primary outcome was reduced both before and after propensity score matching (*P* < 0.0001) (Figure 1B and **Table** **1**). The hazard ratios of both univariate and multivariate analyses confirmed a significant reduction of in‐hospital mortality for the UFH group compared to the control group (Table 1).

**Table 1:** Association between UFH and clinical outcomes in adult patients with sepsis.

Pre‐matched cohort	Control group	UFH group	*P value*	Univariate analysis		Multivariate analysis	
	(n = 4597)	(n = 1928)		Effect size (95% CI)	*P* value	Effect size (95% CI)	*P* value
In‐hospital mortality^a)^, n (%)	794 (17.3%)	310 (16.1%)	0.241	HR = 0.68 (0.60–0.78)	<0.001	HR = 0.68 (0.59–0.77)	<0.001
Length of ICU stay^b)^, days	4.0 (2.8, 7.5)	5.1 (3.1, 9.8)	< 0.001	β = 1.49 (1.16–1.82)	<0.001	β = 1.42 (1.09–1.75)	<0.001
Length of hospital stay^b)^, days	8.8 (5.6, 14.4)	11.8 (7.6, 19.3)	< 0.001	β = 3.94 (3.32–4.56)	<0.001	β = 3.81 (3.19–4.43)	<0.001
Intracranial hemorrhage^a)^	244 (5.3%)	175 (9.1%)	0.002	HR = 1.28 (1.17–1.36)	0.045	HR = 1.23 (1.13–1.28)	0.033
Gastrointestinal bleeding	92 (2.0%)	44 (2.3%)	0.463	HR = 1.13 (0.88–1.27)	0.242	HR = 1.12 (0.79–1.23)	0.313
**Post‐matched cohort**	**Control group**	**UFH group**					
	**(n = 1924)**	**(n = 1924)**					
In‐hospital mortality^a)^, n (%)	361 (18.8%)	308 (16.0%)	0.024	HR = 0.61 (0.53–0.72)	<0.001	HR = 0.63 (0.54–0.74)	<0.001
Length of ICU stay^b)^, days	4.1 (2.8, 7.2)	5.1 (3.1, 9.8)	< 0.001	β = 1.59 (1.18–1.99)	<0.001	β = 1.58 (1.17–1.98)	<0.001
Length of hospital stay^b)^, days	8.7 (5.7, 14.4)	11.9 (7.7, 19.3)	< 0.001	β = 4.12 (3.37–4.87)	<0.001	β = 4.1 (3.36–4.84)	<0.001
Intracranial hemorrhage	164 (8.5%)	172 (8.9%)	0.065	HR = 1.25 (0.88–1.82)	0.083	HR = 1.18 (0.85–1.42)	0.257
Gastrointestinal bleeding	38 (2.0%)	40 (2.1%)	0.814	HR = 1.10 (0.97–1.15)	0.331	HR = 1.05 (0.95–1.10)	0.410

Values are shown as median (interquartile range) or n (%) unless otherwise indicated.

^a)^ A Cox regression model was used to evaluate the impact of UFH on the clinical data after adjusting for age, sex, Sequential Organ Failure Assessment (SOFA) score, and Simplified Acute Physiology Score (SAPS) II. The results are presented as the hazard ratio (HR) and 95% confidence interval (CI).

^b)^ A linear regression model was used to evaluate the impact of UFH use on quantitative clinical data after adjusting for age, sex, SOFA score, and SAPS‐II. Results are presented as the β‐coefficient and 95% CI.

Regarding the secondary outcomes including the length of stay in the ICU and hospital and the risk of bleeding, UFH use was associated with a slightly longer stay in both the hospital and ICU either with or without matching (Figure 1C and Table 1) but UFH use was not associated with the occurrence of either intracranial hemorrhage or gastrointestinal bleeding either with or without matching (Figure 1D and Table 1). In addition, multivariate Cox proportional hazards models further confirmed that the application of UFH was significantly associated with a reduced mortality in all subgroup analyses regardless of age, sex, and severity of organ damage (Figure 1E).

**Figure 2:**
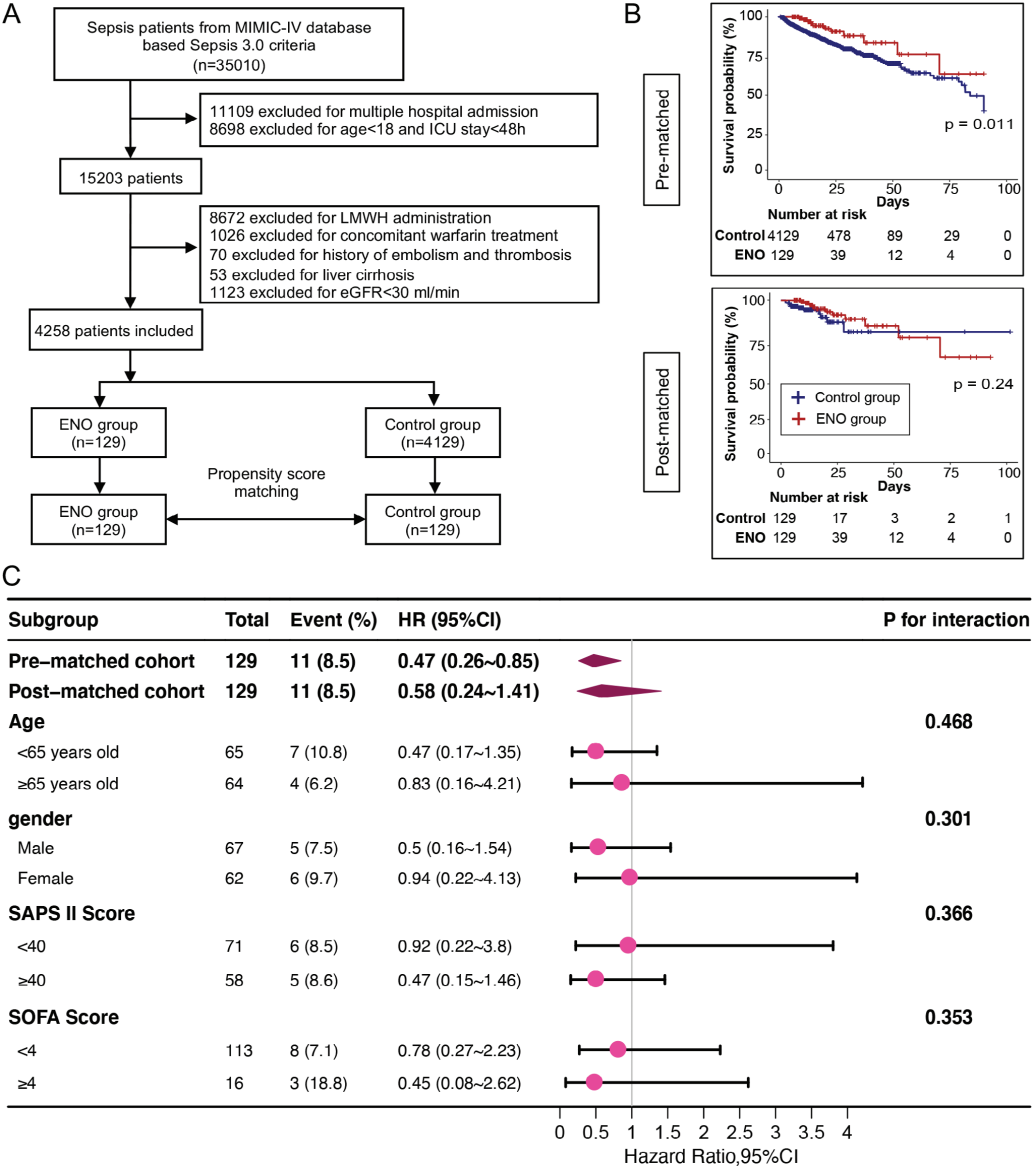
Efficacy of ENO therapy on sepsis outcomes. A) Flowchart illustrating the selection of patients with sepsis from the MIMIC‐IV database. B) Kaplan–Meier survival curves comparing the effect of ENO on the survival rates of patients with sepsis before and after propensity score matching. C) Forest plot of hazard ratios (HRs) for mortality in pre‐ and post‐matched cohorts analyzed by age, sex, Simplified Acute Physiology Score (SAPS) II, and Sequential Organ Failure Assessment (SOFA) score. *P* < 0.05 was considered to indicate a statistically significant effect. LMWH, low‐molecular‐weight heparin; eGFR, estimated glomerular filtration rate.

To investigate whether enoxaparin (ENO), which is also commonly used to prevent and treat disseminated intravascular coagulation in sepsis, has a protective effect on the clinical prognosis of patients with sepsis, a retrospective analysis was conducted with another clinical cohort from the MIMIC‐IV database. After matching propensity scores, 129 patients each were included in the ENO and control groups (**Figure** **2**A) and the clinical characteristics of the study population are summarized in Table S2 (Supporting Information). The Kaplan–Meier curve showed no significant effect of ENO therapy on the survival of adult patients with sepsis after propensity score matching (*P* = 0.24) (Figure 2B and **Table** **2**). Subgroup analysis further confirmed that ENO treatment did not affect the survival prognosis of critically ill patients with sepsis, regardless of age, sex, or severity of organ damage (Figure 2C). These results suggest that although both UFH and ENO can serve as anticoagulants in sepsis treatment, the unique non‐anticoagulant properties of UFH may play a key role in treating sepsis and improving clinical outcomes.

**Table 2:** Association between ENO treatment and clinical outcomes in adult patients with sepsis.

Pre‐matched cohort	Control group	ENO group	*P* value	Univariate analysis		Multivariate analysis	
	(n = 4129)	(n = 129)		Effect size (95% CI)	*P* value	Effect size (95% CI)	*P* value
In‐hospital mortality^a)^, n (%)	457 (11.1)	11 (8.5)	0.364	HR = 0.47 (0.26–0.86)	0.015	HR = 0.47 (0.26–0.85)	0.013
Length of ICU stay^b)^, days	3.9 (2.7, 6.9)	5.2 (3.1, 8.9)	< 0.001	β = 2.76 (1.59–3.93)	<0.001	β = 2.63 (1.47–3.79)	<0.001
Length of hospital stay^b)^, days	9.5 (6.1, 16.6)	16.1 (10.9, 28.5)	< 0.001	β = 8.99 (6.32–11.66)	<0.001	β = 8.73 (6.1–11.36)	<0.001
**Post‐matched cohort**	**Control group**	**ENO group**					
	**(n = 129)**	**(n = 129)**					
In‐hospital mortality, n (%)	11 (8.5)	11 (8.5)	1	HR = 0.6 (0.26–1.41)	0.24	HR = 0.58 (0.24–1.41)	0.231
Length of ICU stay, days	5.0 (2.7, 8.6)	5.2 (3.1, 8.9)	0.154	β = 1.98 (‐0.21–4.16)	0.077	β = 1.7 (‐0.43–3.83)	0.119
Length of hospital stay^b)^, days	10.7 (6.2, 19.5)	16.1 (10.9, 28.5)	< 0.001	β = 8.02 (4.13–11.9)	<0.001	β = 7.23 (3.45–11)	<0.001

Values are shown as median (interquartile range) or n (%) unless otherwise indicated.

^a)^ A Cox regression model was used to evaluate the impact of EMO on the clinical data after adjusting for age, sex, Sequential Organ Failure Assessment (SOFA) score, and Simplified Acute Physiology Score (SAPS) II. The results are presented as the hazard ratios (HR) and 95% confidence intervals (CI).

^b)^ A linear regression model was used to evaluate the impact of EMO use on quantitative clinical data after adjusting for age, sex, SOFA score, and SAPS‐II. Results are presented as β‐coefficient and 95% CI.

### 2.2 UFH Binds to and Inhibits Drp1 in Sepsis to Exert Non‐Anticoagulant Therapeutic Effects

**Figure 3:**
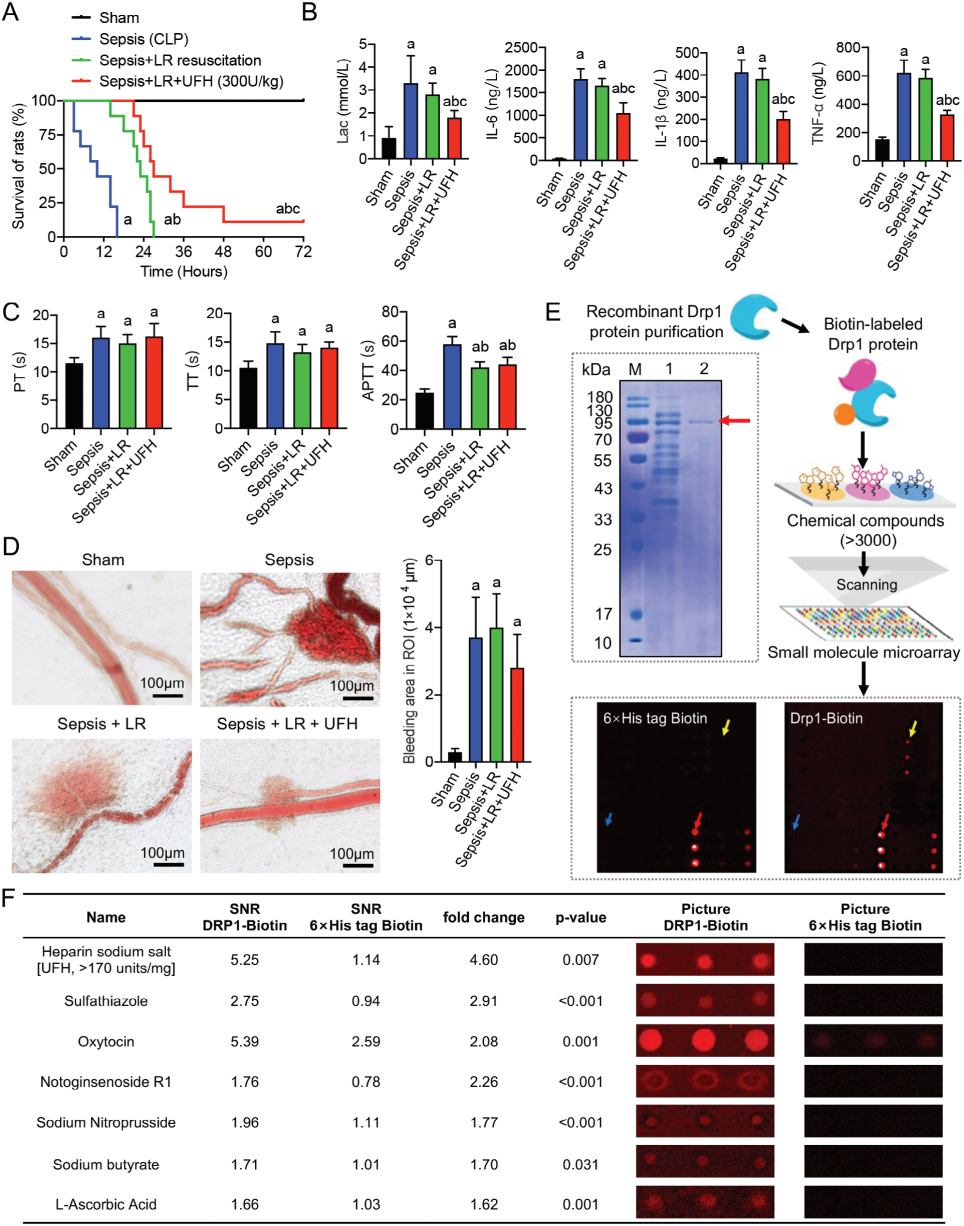
Multifaceted effects of UFH in a septic rat model. A) Effect of UFH on survival rate and duration of sepsis (n = 9 per group) in the cecal ligation and puncture (CLP)‐induced sepsis rat model. B) Comparative analysis of biochemical markers, including lactate, interleukin (IL)‐6, IL‐1β, and tumor necrosis factor (TNF)‐α, after UFH treatment (n = 5 per group). C) Assessment of coagulation parameters, including prothrombin time (PT), thrombin time (TT), and activated partial thromboplastin time (APTT), after UFH treatment (n = 5 per group). D) Microscopic observation of the coagulation dynamics (n = 3). Data are presented as mean ± standard deviation. E) Sodium dodecyl sulfate‐polyacrylamide gel electrophoresis results for purification of recombinant Drp1 (upper left) and the steps for applying small‐molecule microarrays (upper right). The lower panel shows scan images of the control (6× His‐tag biotin) and experimental (Drp1–biotin) samples on the chip. Red arrows indicate the positive control (biotin), blue arrows indicate the negative control (dimethyl sulfoxide), and yellow arrows indicate positive small‐molecule hits. F) Potentially positive small‐molecule analysis results. The fold change was calculated to indicate the extent to which the experimental sample was higher than the control sample, with a threshold of fold change ≥ 1.5 used to identify a potentially specific positive small molecule for the experimental sample. a: *P* < 0.05 compared with Sham group, b: *P* < 0.05 compared with Sepsis (CLP) group. c: *P*<0.05 compared with Sepsis + LR group.

To validate the therapeutic efficacy of UFH for sepsis in vivo, the cecal ligation and puncture (CLP) severe sepsis model was established in rats. Compared to those of the sepsis‐only and sepsis with lactated Ringer's (LR) resuscitation groups, the combined resuscitation with UFH treatment (300 U kg^−1^) significantly prolonged the survival time (*P* < 0.05) (**Figure** **3**A) and significantly reduced blood lactate levels (*P* < 0.05) and inflammatory responses (*P* < 0.05) (Figure 3B), without exacerbating coagulation abnormalities (*P* > 0.05) (Figure 3C) or increasing hemorrhage risk (*P* > 0.05) (Figure 3D). Thus, the treatment efficacy of UFH in sepsis identified in preliminary clinical data analysis (Figure 1) was further verified in in vivo. However, the mechanisms through which UFH achieves the observed therapeutic effects on sepsis remain unclear.

**Figure 4:**
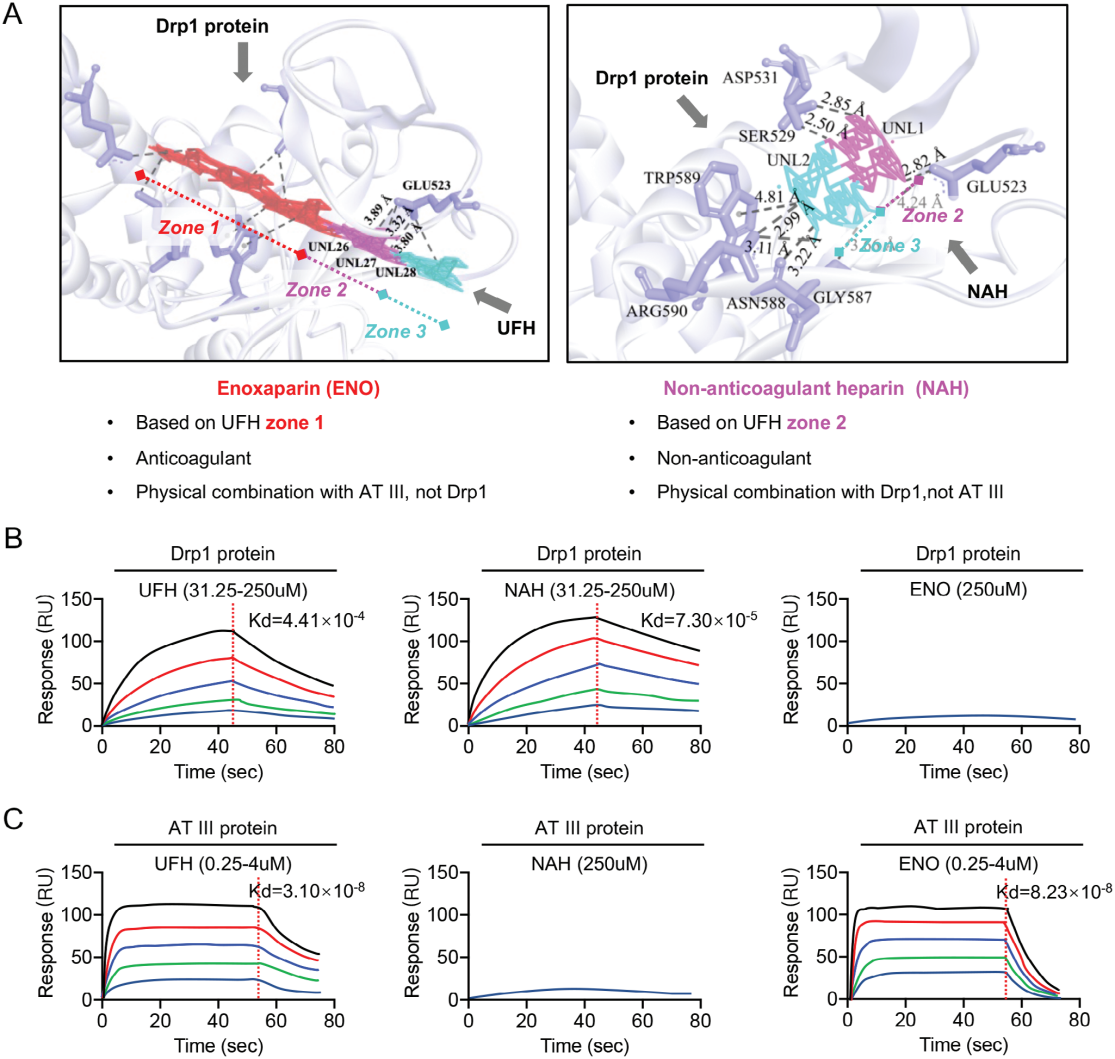
Interaction Analysis of UFH with Drp1. A) Schematic representation of UFH and NAH docking with Drp1. B) Surface plasmon resonance assays depicting the affinity of UFH, NAH, and ENO for Drp1. C) Surface plasmon resonance assays showing the binding affinity of UFH, NAH, and ENO for AT III protein.

Given the critical role of Drp1 in the development and progression of critical illnesses, including sepsis,^[^ ^13^, ^16^ ^]^ we further explored whether the therapeutic action of UFH in sepsis is associated with Drp1, using a purified recombinant Drp1 protein (Figure 3E; Figure S1, Supporting Information). In addition, a small‐molecule microarray chip (Figure 3F; Figure S2, Supporting Information) of Food and Drug Administration (FDA)‐approved drugs, traditional Chinese medicine monomers such as notoginsenoside R1, and small‐molecule inhibitors including Mdivi‐1 potentially targeting Drp1 were screened. The high‐throughput chip‐scanning results suggested potential specific binding between UFH and Drp1 (fold change = 4.60; *P* = 0.007) (Figure 3F; Figure S2A, Supporting Information), but ENO does not have the ability to bind directly to Drp1 (Figure S2A,B, Supporting Information). Chemical structure analysis of UFH revealed three zones, with zone 1 having anticoagulant properties and zones 2 and 3 having non‐anticoagulant properties (Figure S3A, Supporting Information). To identify the specific UFH segment binding to Drp1, a molecular docking model was simulated between UFH and Drp1 using the H‐dock method (Figure S3B,C, Supporting Information), confirming specific binding (docking score = −739.27) (Figure S3D, Supporting Information) and identifying the non‐anticoagulant zone 2 as the binding segment, rather than the anticoagulant zone 1 (**Figure** **4**A). ENO and non‐anticoagulant N‐acetyl heparin sodium (NAH) were selected as representatives of the UFH zone 1 and zone 2 segments, respectively, for molecular docking with Drp1. The results confirmed that ENO did not bind to Drp1, whereas NAH tightly bound to Drp1 at the Glu523 residue (Figure 4A; Figure S3E–G, Supporting Information), thereby mirroring the effect of UFH binding on Drp1.

To validate the molecular docking results, SPR was used to ascertain whether UFH directly binds to Drp1. The results demonstrated that both UFH and NAH bound to Drp1 with high affinity through physical interactions. In contrast, ENO did not bind to Drp1 (Figure 4B). Additionally, both UFH and ENO physically bound to AT III protein, forming complexes that deactivate thrombin and exert anticoagulant effects. NAH, which lacks the anticoagulant segment (zone 1) of UFH, did not bind to AT III (Figure 4C). These results indicated that the effectiveness of UFH in treating sepsis through non‐anticoagulant means is likely due to the specific interaction of the zone 2 non‐anticoagulant segment with Drp1 protein, leading to the suppression of Drp1 functions after sepsis.

### 2.3 UFH Binds to Cytoplasmic Drp1 to Inhibit its Translocation to Mitochondria and Preserve Mitochondrial Quality

During fluid resuscitation in sepsis, UFH primarily interacts with vascular endothelial cells (VECs). Therefore, to explore the impact of the specific binding of UFH to Drp1 on mitochondrial quality in cultured primary VECs after sepsis, UFH was initially tagged with fluorescein isothiocyanate (FITC) to track its cellular penetration and localization (Figure S4A, Supporting Information); the absence of free FITC–NH2 in the product was verified (Figure S4B, Supporting Information), indicating successful synthesis of the FITC–UFH product. Confocal microscopy images demonstrated that UFH was capable of entering cells and colocalized with cytoplasmic Drp1 (Figure S4C, Supporting Information), indicating that UFH not only acts on cell membrane receptors^[^ ^17^ ^]^ but also penetrates into cells, exerting its effects through specific binding to cytoplasmic Drp1.

**Figure 5:**
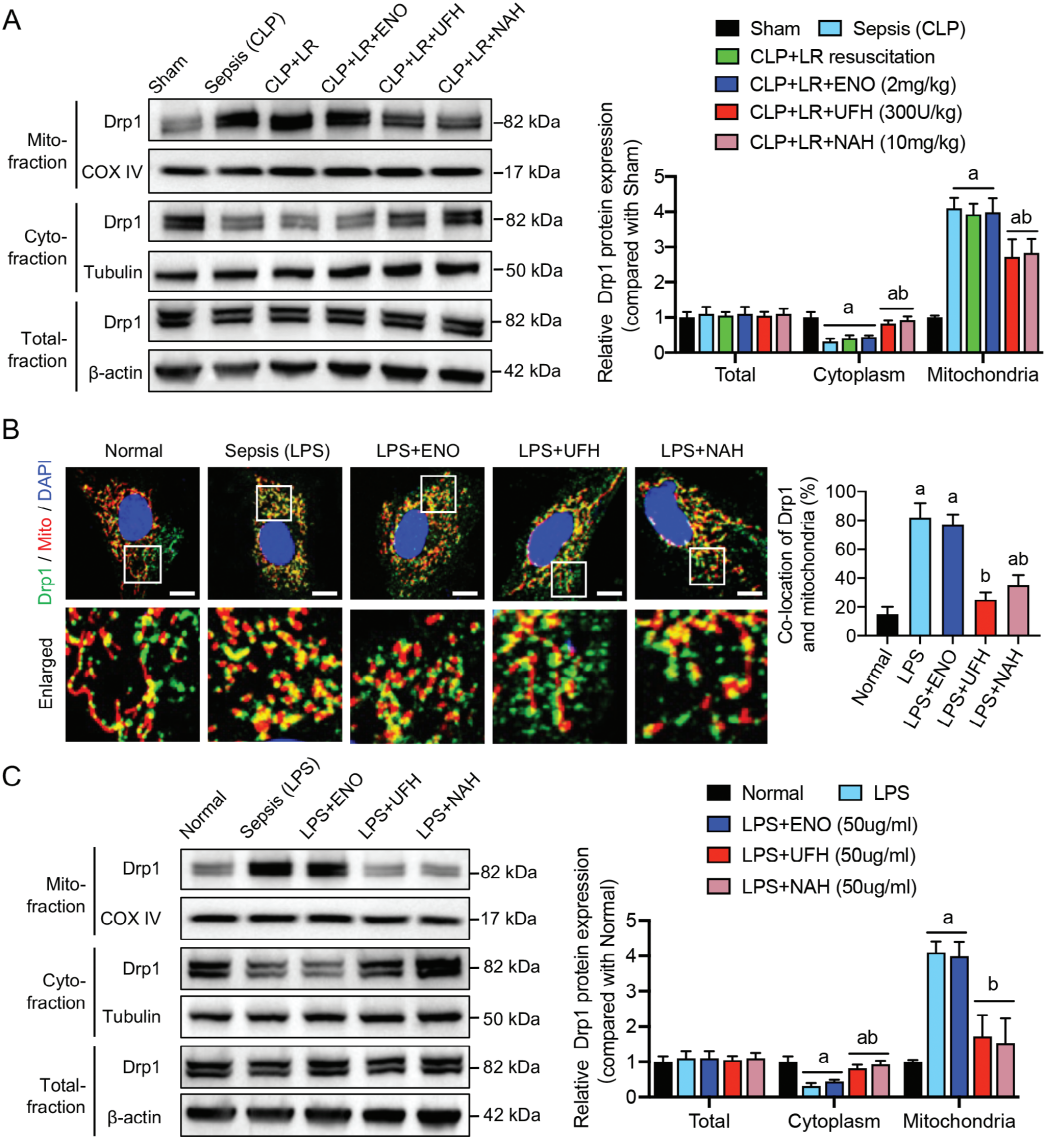
Drp1 localization in vascular endothelial cells (VECs) under sepsis. A) Drp1 expression in the blood vessels of rats from each group (n = 3). B) Confocal images and quantification of Drp1‐ and MitoTracker‐labeled mitochondria in lipopolysaccharide (LPS)‐treated VECs as an in‐vitro sepsis model; scale bars, 20 µm (n = 3). C) Drp1 expression in the subcellular regions of VECs in each group (n = 3). Lowercase letters a and b above bars indicate a significant difference (*P* < 0.05) compared with the Sham or Normal group and compared with the CLP or LPS group, respectively.

To investigate the specific effects of UFH upon entering cells and binding to cytoplasmic Drp1, VECs were collected from the rats with sepsis and separated their mitochondrial and cytoplasmic components using ultracentrifugation. A significant increase of the mitochondrial Drp1 expression and a decrease in cytoplasmic Drp1 expression in VECs after sepsis, suggesting substantial translocation of Drp1 from the cytoplasm to the mitochondria (**Figure** **5**A). Treatment with UFH and NAH targeting Drp1 significantly reduced mitochondrial Drp1 levels (*P* < 0.05), whereas treatment with LR alone or with ENO did not affect Drp1 translocation (*P* > 0.05) (Figure 5A). At the cellular level, VECs simulated with lipopolysaccharide (LPS) exhibited a significant increase in Drp1 colocalization within the mitochondria (*P* < 0.05). UFH or NAH treatment led to ≈50% reductions in Drp1–mitochondrial colocalization (*P* < 0.05), whereas ENO treatment did not significantly alter this colocalization (*P* > 0.05) (Figure 5B; Figure S4D, Supporting Information). Western blot data with samples derived from rats (Figure 5C) were corroborated with these confocal images (Figure 5C). Thus, these results obtained from both cells and animals confirmed that UFH binds to cytoplasmic Drp1, thereby diminishing the aggregation of cytoplasmic Drp1 moving into the mitochondria following sepsis.

**Figure 6:**
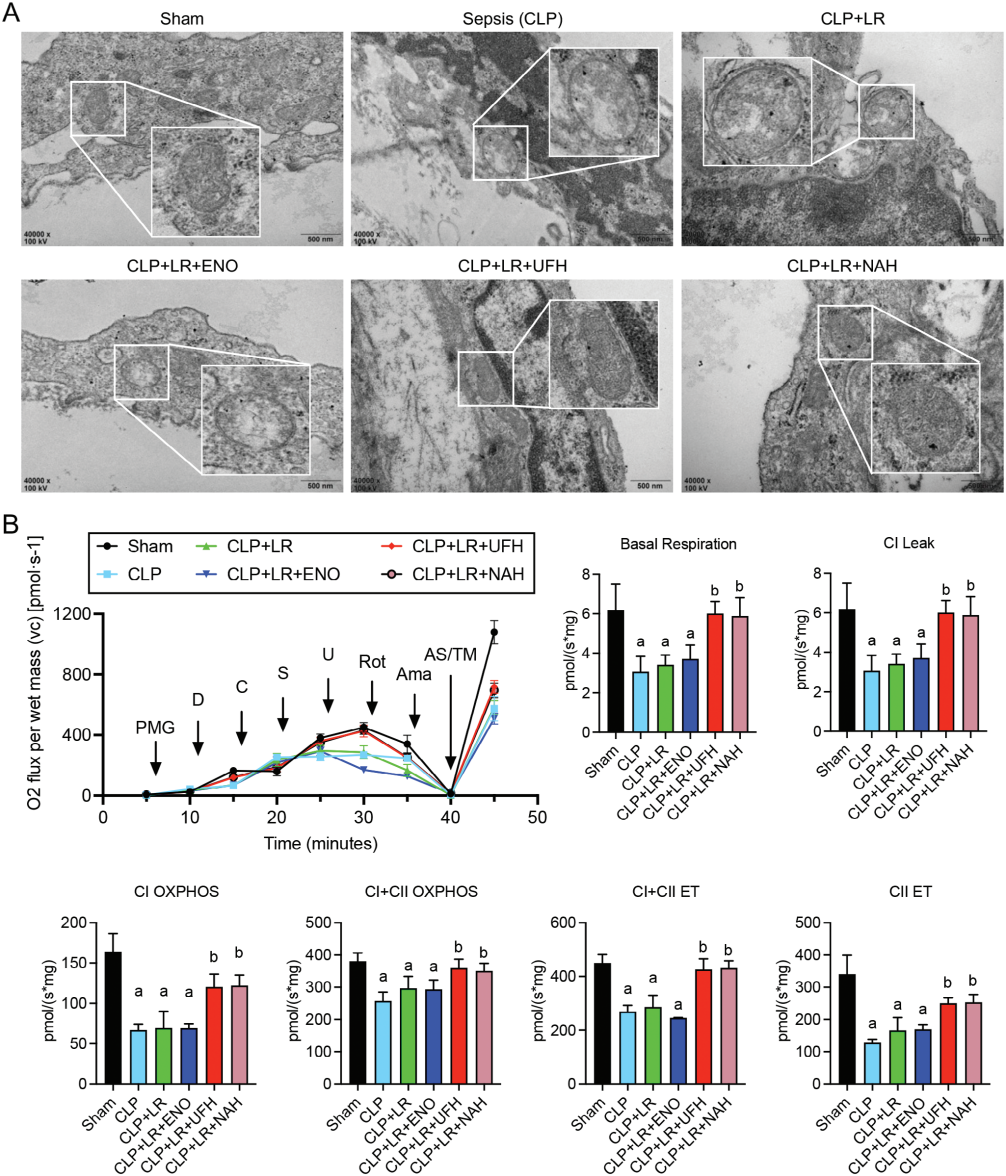
Comparative effects of ENO, UFH, and NAH treatments on mitochondrial quality of the vasculature in sepsis. A) Electron microscopy images of mitochondrial morphology in the vessel tissues of control and cecal ligation and puncture (CLP)‐induced septic rats with different treatments; scale bars, 500 nm (n = 3). B) Representative images of O2 flux per mass in each group (top left). The summarized data of the oxygen consumption capacity, as measured by high‐resolution respirometry in complex I and II, including CI leak, CI P (OXPHOS), CI + CII P, CI + CII ETS (electron transfer system capacity), and CII ETS (n = 3). Lowercase letters a and b above indicate a significant difference (*P* < 0.05) compared with the Sham group and CLP group, respectively.

Transmission electron microscopy observations further revealed a significant mitochondrial quality imbalance in the VECs harvested from rats with sepsis, characterized by extensive vacuolation and disrupted cristae structures. These mitochondrial abnormalities, including extensive vacuolization, were still evident after LR treatment or combined resuscitation with ENO. However, treatment with UFH or NAH notably improved the integrity of the mitochondrial cristae and reduced the extent of vacuolization (**Figure** **6**A). In addition, we used the Oroboros Oxygraph‐2k to assess mitochondrial respiration. Compared to the Sham group, CLP treatment significantly reduced mitochondrial OXPHOS capacity and the activity of the electron transport system (ETS) of complex I. This was evident in the decreased CI leak, CI and CI+CII OXPHOS, CI+CII ETS and CII ETS. However, these changes were significantly reversed in rats treated with heparin, whereas the changes were not notable in those treated with ENO (Figure 6B). This suggests that the specific binding of UFH to Drp1 enhanced the mitochondrial quality in vascular endothelial cells (VECs) following sepsis. (Figure 6B), suggesting that the specific binding of UFH to Drp1 enhanced the mitochondrial quality in VECs after sepsis.

**Figure 7:**
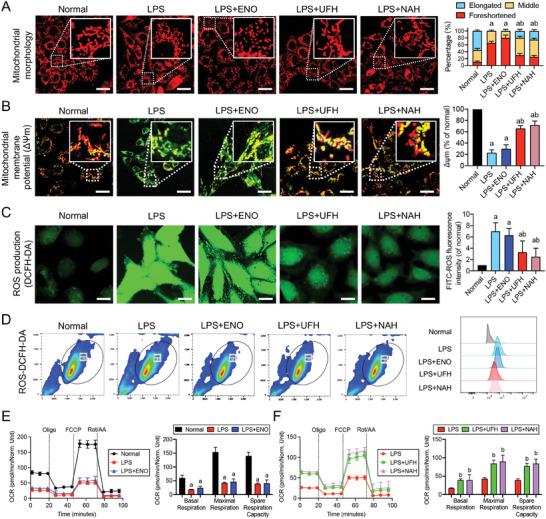
Comparative effects of ENO, UFH, and NAH treatments on mitochondrial quality of vascular endothelial cells (VECs) in sepsis. A) Representative confocal microscopy images of mitochondria in VECs (scale bars, 50 µm). B) Representative confocal microscopy images of mitochondrial membrane potential (ΔΨm); scale bars, 50 µm (n = 3). C) Representative confocal microscopy images of reactive oxygen species (ROS) production, measured according to the fluorescent intensity, in VECs; scale bars, 30 µm (n = 3). D) Detect the ROS level in each group using flow cytometry in VECs (n = 3). E) Effect of heparin on mitochondrial respiration in VECs (n = 3). Lowercase letters a and b above indicate a significant difference (*P* < 0.05) compared with the normal group and LPS group, respectively.

We further explored the detailed impact of the different treatments on mitochondrial morphology and function at the cellular level. Confocal microscopy images showed that LPS‐stimulated VECs exhibited fragmented mitochondrial structures. Notably, ENO treatment did not significantly ameliorate this fragmentation (*P* > 0.05) and even exacerbated the damage in some cases. In contrast, UFH or NAH treatments effectively restored mitochondrial morphology in VECs, significantly transforming the fragmented structures into filamentous or short columnar shapes (*P* < 0.05) (**Figure** **7**A). These results confirmed that the specific binding of UFH to Drp1 improves mitochondrial morphology in VECs after sepsis. Moreover, LPS‐stimulated VECs showed a significant reduction in mitochondrial membrane potential (*P* < 0.05) and a significant increase in reactive oxygen species (ROS) accumulation (*P* < 0.05). ENO treatment did not markedly enhance mitochondrial function (*P* > 0.05), whereas UFH and NAH treatments restored the LPS‐reduced mitochondrial membrane potential (*P* < 0.05) (Figure 7B; Figure S5, Supporting Information) and reduced the level of ROS production (*P* < 0.05) (Figure 7C,D). Collectively, these results indicated that the specific binding of UFH to Drp1 improved mitochondrial function in VECs after sepsis.

Seahorse X24 analysis further indicated mitochondrial metabolic dysfunction in LPS‐stimulated VECs, characterized by a substantial decrease in the mitochondrial oxygen consumption rate (OCR). ENO treatment did not improve the mitochondrial OCR (*P* > 0.05) (Figure 7E) but UFH or NAH treatments significantly enhanced various mitochondrial metabolic parameters, including basal respiration, maximal respiration, and spare respiratory capacity (*P* < 0.05) (Figure 7F). These results demonstrated that specific binding of UFH to Drp1 improves mitochondrial metabolism profile in VECs after sepsis.

### 2.4 Targeted Inhibition of Drp1 by UFH Reduces Vascular Leakage and Enhances Prognosis in Sepsis

**Figure 8:**
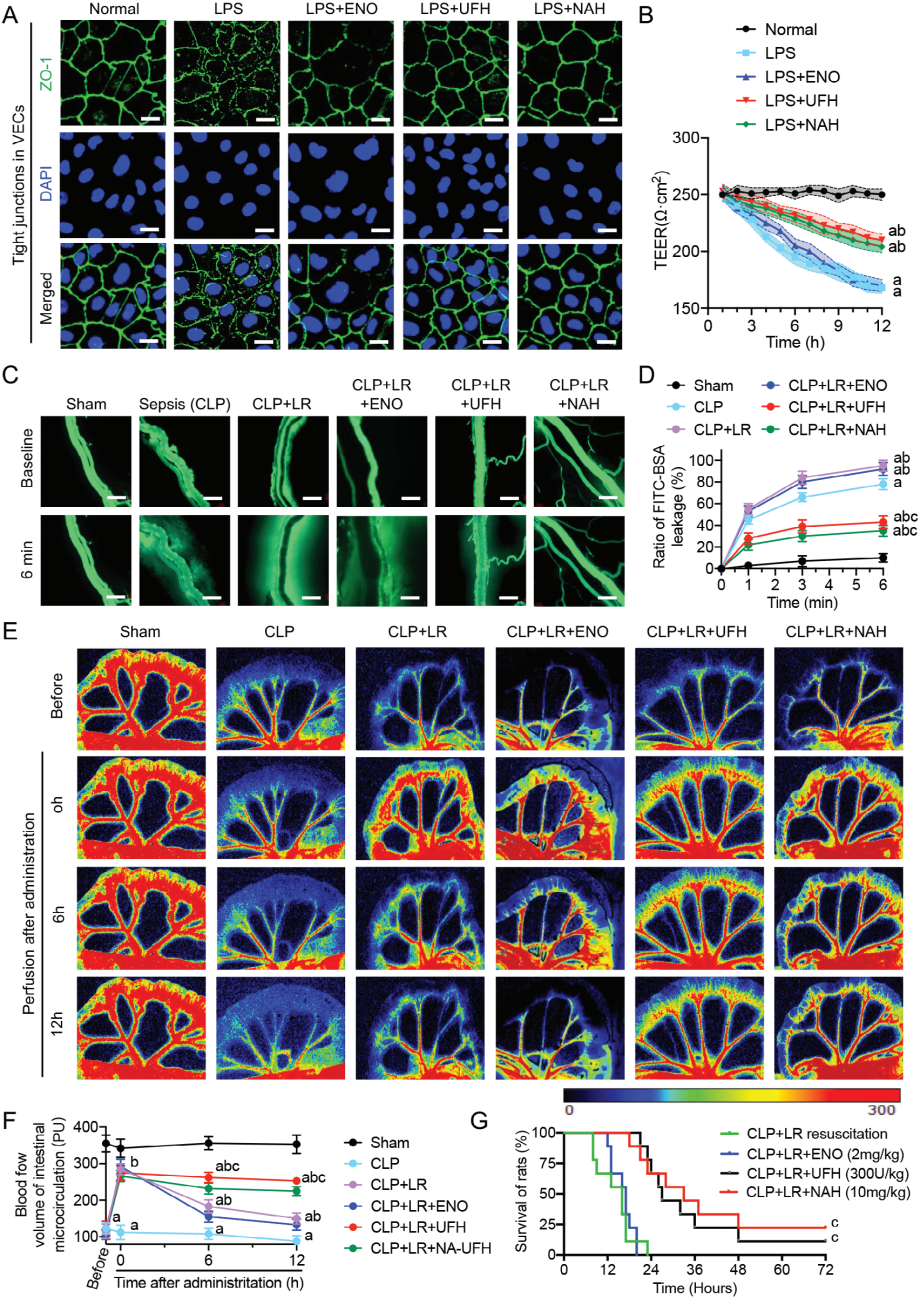
Differential impacts of ENO, UFH, and NAH on vascular endothelial integrity and prognosis in the rat sepsis model. A) Immunofluorescent detection of tight junctions (ZO‐1) in vascular endothelial cells (VECs) across treatment groups; scale bars = 50 µm (n = 3). B) Transmembrane electrical resistance (TEER) measurements of VEC monolayers in each group. C) FITC–BSA permeability in mesenteric microvessels; scale bars = 20 µm (n = 3). D) Quantitative analysis of vascular leakage. E) Speckle tomography visualization of mesenteric blood flow (n = 3). F) Quantification of mesenteric perfusion rates. G) Survival analysis after treatment with ENO, UFH, or NAH in rats with sepsis (n = 9). Lowercase letters a, b, and c above indicate a statistically significant difference (*P* < 0.05) compared with the Sham or Normal group, compared with the CLP or LPS group, and compared with the LR group, respectively.

To further explore the effects of UFH on vascular endothelial function, cultured primary VECs were challenged with LPS with or without NEO, UFH or NAH treatment. Confocal microscopy showed that the integrity of the tight junctions between VECs (labeled for the marker ZO‐1) was disrupted following LPS stimulation. Treatment with ENO did not significantly improve this disruption, whereas treatment with UFH or NAH restored the tight junctions (**Figure** **8**A). Additionally, Transwell assays confirmed that UFH and NAH notably decreased the permeability of LPS‐stimulated VECs (*P* < 0.05) (Figure 8B), underscoring the non‐anticoagulant properties of UFH in maintaining vascular endothelial integrity after sepsis.

To further assess the effect of UFH treatment on vascular permeability in sepsis in vivo, its effects on mesenteric microvascular permeability were evaluated in the rat model. Microcirculatory fluorescence recordings showed that the permeability of mesenteric microveins to FITC‐labeled bovine serum albumin (BSA) was significantly increased after sepsis (*P* < 0.05). Resuscitation with LR alone or ENO treatment failed to reduce the permeability and instead exacerbated the degree of vascular leakage (*P* < 0.05). In contrast, treatment with UFH or NAH significantly reduced the permeability of microveins (*P* < 0.05) (Figure 8C,D; Figure S6, Supporting Information).

To further verify its effect of UFH on vascular integrity, microcirculatory blood perfusion in rats was evaluated using a laser speckle blood flow imaging system. Mesenteric microcirculatory blood flow was severely compromised in rats with sepsis (*P* < 0.05). Although resuscitation with LR alone or in combination with ENO rapidly expanded the blood volume and restored blood flow, maintaining microcirculatory perfusion over time with an ≈50% reduction in perfusion detected at 6 h after resuscitation compared to that recorded immediately after resuscitation. In contrast, treatment with UFH or NAH successfully maintained microcirculatory perfusion over time (*P* < 0.05) (Figure 8E,F). Survival analysis demonstrated that both NAH or UFH but not ENO enhanced the survival (*P* < 0.05) (Figure 8G). These outcomes are consistent with the clinical data (Figures 1 and 2), suggesting that UFH can bind to cytoplasmic Drp1, reducing its translocation to mitochondria. This interaction mitigates the subsequent Drp1‐mediated mitochondrial quality imbalance, which positively affects the functionality of endothelial tight junctions and vascular permeability.

## 3 Discussion

**Figure 9:**
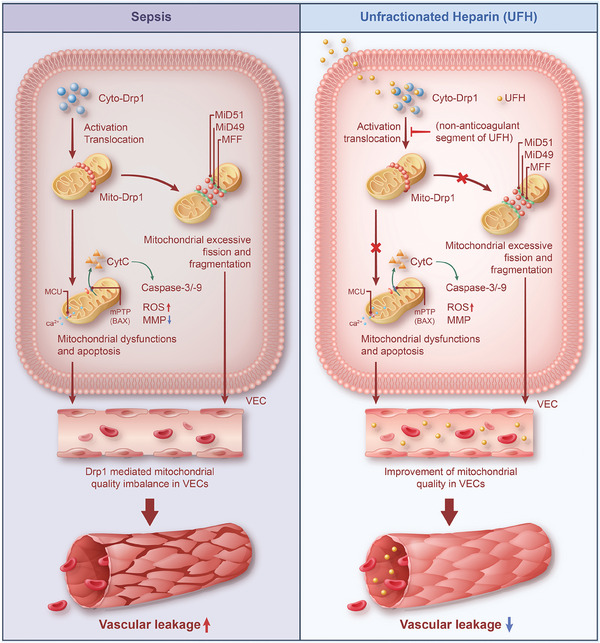
Schematic of the non‐anticoagulant mechanism of unfractionated heparin in ameliorating sepsis outcomes by modulating mitochondrial quality.

Our study found that the zone 2 non‐anticoagulant segment of UFH specifically binds to Drp1, inhibiting its modification activity and mitochondrial translocation. Subsequently, UFH inhibits Drp1 through non‐anticoagulant mechanisms, improves mitochondrial quality in vascular endothelial cells, reduces vascular leakage, and ultimately attenuates lung injury and enhances survival of septic rats (**Figure** **9**). This study sheds a light on the non‐anticoagulant mechanism of UFH in treating sepsis through targeting Drp1‐mediated mitochondrial quality imbalance and provides insights for developing non‐anticoagulant heparin derivatives for organ protection in critical illness settings.

Appropriate anticoagulation therapy is recognized as the key strategy for preventing microthrombus formation and slowing sepsis disease progression.^[^ ^18^ ^]^ In the clinical setting, UFH and ENO are commonly used as anticoagulants. Although some studies suggested that heparin does not improve the 28‐day survival rate of patients with sepsis,^[^ ^19^ ^]^ a considerable amount of studies highlighted its positive effect on sepsis outcomes.^[^ ^20^, ^21^ ^]^ A retrospective analysis, refined by rigorous propensity matching, further substantiated that appropriate heparin administration in patients with sepsis did not increase the risk of bleeding and substantially lowered mortality rate. However, ENO did not demonstrate similar organ‐protective effects against sepsis as UFH seen in our study albeit in the cell level and pre‐clinical setting. In line with our findings, Peng et al.^[^ ^22^ ^]^ showed a significant reduction in the 28‐day mortality and in‐hospital death rate in patients with sepsis treated with UFH but without an increased risk of intracranial or gastrointestinal bleeding. Although another previous study claimed the effectiveness of ENO in reducing sepsis mortality,^[^ ^23^ ^]^ this analysis may have been skewed due to inadequate propensity score matching, which may distorted the results due to the interference of factors such as patient age and Sequential Organ Failure Assessment (SOFA) scores.

UFH is a glycosaminoglycan sulfate derived and refined from the pig intestinal mucosa or bovine lungs, with a molecular weight ranging from 3000 to 30 000 kDa. UFH primarily comprises three zones, among which zone 1, a pentasaccharide sequence, mainly binds to AT III to exert its anticoagulant effects.^[^ ^10^ ^]^ ENO, a low‐molecular‐weight heparin product, was synthesized from UFH anticoagulant zone 1. However, the molecular docking and SPR affinity testing results revealed that the optimal binding region of UFH for Drp1 lies in its non‐anticoagulant zone 2 (residues U26–U28). In contrast, ENO lacks the capacity to specifically bind Drp1. Our study demonstrated that the non‐anticoagulant zone 2 of UFH specifically binds to Drp1 protein, thereby inhibiting its function following sepsis. This finding may explain why UFH is significantly effective in sepsis treatment, whereas other anticoagulants such as ENO have limited effects. Furthermore, Lv et al. reported competitive binding between UFH and LPS at the cell membrane surface,^[^ ^17^ ^]^ while Shao's research indicates that LPS can enter the cell interior via the LBP‐CD14 transport/endocytosis pathway.^[^ ^24^ ^]^ Based on these observations, we hypothesized that the competitive binding of UFH with LPS at the cell membrane might also occur inside the cell, potentially influencing mitochondrial quality regulation mediated by Drp1. Through in vitro and in vivo experiments, we confirmed that UFH indeed binds to Drp1 within the cell. The discovery of this intracellular mechanism not only provides a new perspective on the therapeutic potential of UFH beyond its traditional anticoagulant role but also encourages further investigation into the specific molecular interactions between UFH and mitochondrial proteins.

In sepsis, mitochondrial quality undergoes significant changes, including excessive mitochondrial fission, reduced mitochondrial respiration, increased oxidative stress, and decreased mitochondrial membrane potential.^[^ ^25^ ^]^ Drp1 is a member of the GTP‐binding dynamin family and a key regulator of mitochondrial fission. It is translocated from the cytoplasm to the outer mitochondrial membrane, where it assembles into helical oligomers that encircle the mitochondria, mediating constriction and division. Post‐sepsis alterations in Drp1 activity and protein binding are crucial factors contributing to pathological mitochondrial fission and dysfunction.^[^ ^13^ ^]^ Accordingly, the inhibition of Drp1 activity and its protein‐binding function alleviated cell oxidative stress mediated by sepsis and maintain membrane potential, significantly protecting organ function.^[^ ^13^ ^]^ Otherwise, the improvement in mitochondrial quality was found to be closely associated with the enhancement of tight junction functions between endothelial cells. This association underlies a crucial mechanism in which better mitochondrial health supports the integrity of endothelial barriers, reducing the occurrence of vascular leakage. Numerous studies confirm that better mitochondrial integrity significantly improves the stability and functionality of endothelial tight junctions, thereby mitigating vascular leakage in conditions like sepsis.^[^ ^26^, ^27^ ^]^ This study demonstrates that the binding mechanism between UFH and Drp1 primarily involves direct interaction between the non‐anticoagulant region (Zone 2) of UFH and the Glu 523 site on Drp1. This interaction reduces the translocation of cytoplasmic Drp1 to the mitochondria, thereby improving mitochondrial function and morphology. This finding underscores the importance of protecting mitochondrial quality in enhancing sepsis prognosis through a non‐anticoagulant pathway. Furthermore, this study indicated that UFH‐targeted inhibition of Drp1‐mediated mitochondrial quality imbalance reduces vascular leakage and prolongs post‐sepsis survival time, which may be of great clinical significance.

Although this study provides valuable insights, it had certain limitations that should be acknowledged and can inform further study designs. First, although CLP‐ model and LPS‐induced endothelial cell injury model were employed to simulate sepsis and explore the therapeutic mechanisms, these models are not entirely representative of the complex pathophysiological environment of acute lethal sepsis in humans. Therefore, more extensive and comprehensive assessment is needed. Second, considering the pivotal role of lung vascular endothelial barrier dysfunction in the progression of sepsis, this study primarily focused on the effects of heparin‐like drugs on VECs and their functions. Given that sepsis is multi‐organ related systemic disease, whether UFH has favorable effects on other cell types such as in the heart and liver as in the lung cells remains unknown. In addition, the specific mechanisms by which UFH penetrates the cell membrane to enter the cytoplasm, such as whether it enters the cell through an endocytosis pathway similar to LPS, and how it affects the activity of Drp1 by binding to it in the cytoplasm, thereby reducing its translocation to the mitochondria, still require further investigation. Despite these limitations, the findings of this study provide a new understanding of the role of UFH in treating sepsis through non‐anticoagulant pathways, and offers novel insights into the developing drugs that target on Drp1‐mediated mitochondrial quality imbalance and improves sepsis outcomes.

## 4 Conclusion

This study provides new insights of UFH in sepsis treatment through non‐anticoagulant pathways. Proper UFH use during sepsis can improve mitochondrial quality in VECs by specifically binding to cytoplasmic Drp1, ultimately reducing vascular leakage and enhancing sepsis prognosis. This finding highlights the potential of UFH as a key component in sepsis treatment strategies.

## 5 Experimental Section

### Animals and Materials

Male Sprague–Dawley rats, aged 9–10 weeks and weighing 200–220 g, were obtained from Chongqing Medical University Experimental Animal Center. The experimental protocol was followed with the ARRIVE guideline and was approved by the Ethics Committee of Chongqing Medical University, Chongqing, China (No. SYXK‐2023‐0005‐2022‐222).

Unfractionated heparin sodium (UFH, H3149), N‐acetyl heparin sodium (NAH, A8036), endothelial cell growth supplement (E2759) and LPS *(Escherichia coli serotype O55:B5)* were purchased from Sigma–Aldrich (St. Louis, MO, USA). Enoxaparin sodium (ENO, S5415) was purchased from Selleck (Shanghai, China). Goat anti‐mouse secondary antibody (A0216), type II collagenase (ST2303‐100 mg), RIPA buffer (P0013B), protease inhibitors (P1005), phosphatase inhibitors (P1045), pro‐light HRP chemiluminescent detection reagent (P0018S), JC‐1 mitochondrial membrane potential fluorescence probe (C2006) and ROS detection solution (S0033S) were purchased from Beyotime (Shanghai, China). Mitochondrial isolation kit (MP‐007) were purchased from Invent Biotechnologies (Beijing, China). Antibodies for β‐Actin (ab8226), COX4 (ab110272), and β‐tubulin (ab7291) were purchased from Abcam (Cambridge, UK). Antibody for Drp1 (4E11B11) was purchased from Novus (Colorado, USA). LR solution was purchased from Zeye Biotechnology (Shanghai, China). Bicinchoninic acid protein assay kit (23 227), high‐glucose Dulbecco's modified Eagle medium (DMEM, 11 965 092), MitoTracker (A66440), Antibody for ZO‐1 (33‐9100) and goat anti‐mouse Alexa Fluor 488 secondary antibody (A‐11001) were purchased from Thermo Fisher Scientific (Waltham, MA, USA). Pyruvate (103575‐100) and glutamine solution (103579‐100), XFe cell culture plate (103025‐100) and mitochondrial stress kit (103260‐100) were purchased from Agilent (Santa Clara, CA, USA). All other chemicals were purchased from Sigma unless specifically mentioned otherwise.

### Clinical Sepsis Patients Cohort Preparation

Clinical data of sepsis patients in the ICU were included for retrospective analysis using the public MIMIC‐IV (version 2.0) real‐word database from The Beth Israel Deaconess Medical Center, including data collected between 2008 and 2019.^[^ ^28^ ^]^ The inclusion criteria were: 1) being 18 years or older and 2) meeting the Sepsis 3.0 definition.^[^ ^29^ ^]^ The exclusion criteria were: 1) an ICU stay of less than 48 h, 2) the use of low‐molecular‐weight heparin or warfarin, 3) thrombosis or embolism history, 4) cirrhosis in decompensation, and 5) insufficient renal function according to an estimated glomerular filtration rate < 30 mL min^−1^·1.73 m^−2^. Only the first ICU admission was considered for patients who had recurrent stays in the ICU.

### Clinical Outcomes and Data Extraction

The primary outcome was in‐hospital mortality rate. Secondary outcomes were length of hospital stay and length of ICU stay. The use of UFH was defined as having a record of subcutaneous or intravenous infusion at preventive or therapeutic doses for at least 5 days in the “prescription in MIMIC‐IV” field. In contrast, patients in the control group had no anticoagulant therapy for 5 days or no anticoagulant therapy at all. Bleeding complications, including intracranial and gastrointestinal bleeding, were stratified according to the International Classification of Diseases, 10th edition. The following variables were extracted from the database: demographic characteristics, vital signs, laboratory test results, Simplified Acute Physiology Score (SAPS) II, SOFA score, and comorbidities.

### Animal Model Establishment and Treatment

The sepsis rat model was established using cecal ligation and puncture (CLP) as previously described.^[^ ^30^ ^]^ Rats were anesthetized with an intraperitoneal injection of pentobarbital sodium (45 mg kg^−1^). The cecum was exteriorized, ligated, and punctured 0.7 cm from the distal end using a triangular needle with an approximate diameter of 1.5 mm, allowing fecal matter to freely enter the abdominal cavity. The omentum on both sides was removed, and the wound was sutured. Postoperative analgesia was administered via subcutaneous injections of buprenorphine (0.05 mg kg^−1^) every 6 h for 2 days.

The rats were randomly divided into six groups: Control, Sepsis (CLP), Resuscitation (CLP + LR solution), ENO (CLP + LR solution + ENO), UFH (CLP + LR solution + UFH), and NAH (CLP + LR solution + NAH). In the Control group, cecal ligation was performed without puncturing, whereas all other procedures remained in identical. The Resuscitation group received 35 mL kg^−1^ of LR solution via femoral vein cannulation 12 h after model establishment, with the infusion completed within 2.5 h. The ENO, UFH, and NAH groups were treated with 2 mg kg^−1^ ENO, 300 U kg^−1^ UFH, or 10 mg kg^−1^ NAH in the LR solution, respectively. In the Sepsis group, ligation was performed followed by femoral vein cannulation. The upper limit for survival was set at 3 days. 12 h after treatment, vascular endothelial tissues were collected for subsequent experiments.

### Cellular Model Establishment and Treatment

Primary vascular endothelial cells (VECs) were harvested from the ileocecal mesenteric veins of Sprague–Dawley rats. After anesthesia and disinfection with iodine, the thoracic cavity was incised, and the ileocecal mesenteric vein was isolated. The vascular tissues were sectioned into 1 mm × 1 mm pieces, digested sequentially with 0.25% trypsin and 2% type II collagenase, and filtered through a 400‐mesh sieve to obtain a cell suspension. The harvested cells were resuspended in DMEM culture medium containing 20% fetal bovine serum, 0.05 mg mL^−1^ endothelial cell growth supplement, and 1% penicillin‐streptomycin mixture. They were then cultured in an incubator at 37 °C with 5% CO_2_. 4 h post‐adhesion, non‐adherent and slowly adherent cells were removed, and the remaining cells were cultured. Primary VECs were obtained from these adherent cells.

The cultured cells were treated as follows: 1) Normal group, cells cultured in DMEM; 2) LPS group, cells treated with 10 µg mL^−1^ LPS in DMEM for 24 h to simulate a septic cell state; 3) LPS+ENO group, LPS‐induced VECs treated with 50 µg mL^−1^ ENO for 12 h;^[^ ^17^, ^31^ ^]^ 4) LPS+UFH group, LPS‐induced VECs treated with 50 µg mL^−1^ UFH for 12 h;^[^ ^17^, ^31^ ^]^ and 5) LPS+NAH group, LPS‐induced VECs treated with 100 µg mL^−1^ NAH for 12 h.^[^ ^17^ ^]^

### Small‐Molecule Microarray Screening

We developed a small‐molecule microarray chip as reported^[^ ^32^ ^]^ for high‐throughput screening of potential Drp1‐targeting FDA‐approved drugs, traditional Chinese medicine monomers, and small‐molecule inhibitors, following the published small‐molecule microarray protocol.^[^ ^32^ ^]^ Initially, 3000 small molecules were selected from an FDA‐approved database and γ‐aminopropyl silane microscope slides were functionalized with Fmoc‐protected amino polyethylene glycol spacers. After deprotection, 1,6‐diisocyanatohexane was coupled onto the slide surface via urea bond formation to prepare functionalized isocyanate‐coated microarray slides (GeneChem, Shanghai, China). These slides, capable of reacting with primary alcohols and amines, formed a fixed chemical screening library.

The selected small molecules, stored in 10 mM dimethyl sulfoxide, were precisely printed on slides and treated with pyridine vapor to facilitate covalent attachment. The slides were then incubated and washed with a polyethylene glycol:dimethyl formamide solution.^[^ ^33^ ^]^ The prepared slides were then incubated with biotin‐labeled Drp1 recombinant protein (KMD Biosciences) and biotin‐labeled 6×His tag carrier protein (5 µg mL^−1^) solutions to screen for small molecules interacting with Drp1. Incubation with Cy5‐labeled streptavidin for 1 h enabled the detection of small molecules bound to biotin‐labeled proteins via fluorescence labeling. After incubation, the slides were washed, dried, and scanned using a LuxScanTM 10 K‐A microarray scanner (Boao Bio, Beijing, China). Data extraction and analysis were performed using GenePix Pro v6.0 software (Molecular Devices, Sunnyvale, CA, USA). Potential positive hits were identified based on fold change ≥1.5 and statistical significance (*P* < 0.05).

### Molecular Docking

Molecular docking of the Drp1 protein with small molecules was performed using H‐dock software. Structural information for the small molecules was obtained from the PubChem database (https://pubchem.ncbi.nlm.nih.gov/compound). Homologous sequences for both the receptor and ligand were identified through sequence similarity searches in the Protein DataBank database, and appropriate templates were selected. 3D structural modeling was conducted using MODELLER software with sequence alignment performed by ClustalW. After completing the structural model, global molecular docking was carried out, and the optimal model was selected based on docking scores and active site information. The docking results were visualized using Discovery Studio software.

### SPR Affinity Detection

The Octet R8 system was employed to assess the affinity between UFH, NAH, ENO, and Drp1. Drp1 was biotinylated for specific adherence to NTA sensor chips. UFH, NAH, and ENO were continuously diluted in the running buffer to obtain a range of concentrations (1–0.03125 mM) and sequentially introduced to the chip for interaction monitoring. An initial 120‐s baseline period was set for equilibrium, followed by 45‐s intervals for the baseline, association, and dissociation phases.^[^ ^31^ ^]^ Data analysis was performed using ForteBio Data Analysis software (version 12.0) with sensor selection, baseline correction, and a 1:1 binding model for curve fitting to elucidate the kinetic data.

### Western Blotting

Tissues, cells, or subcellular components were lysed in RIPA buffer supplemented with complete protease and phosphatase inhibitors. Samples underwent electrophoresis and were transferred onto polyvinylidene fluoride membranes. These membranes were incubated with primary antibodies against Drp1, followed by secondary antibody. Protein concentrations were determined using a bicinchoninic acid protein assay kit. Immunoblotted proteins were visualized using pro‐light HRP chemiluminescent detection reagent. Band intensity was analyzed with Quantity One V 4.62 software (Bio‐Rad, Life Science, USA).β‐Actin, COX4, and β‐tubulin antibodies were used as internal controls for total protein, mitochondrial, and cytosolic fractions, respectively. The relative expression levels of Drp1 were quantified by calculating the ratio of Drp1 to internal control densitometry values using ImageJ software (https://fiji.sc/).

### Immunofluorescence

VECs were seeded in confocal culture dishes and allowed to reach 50% confluence. Depending on the experiment, either diluted MitoTracker (1:10000) or FITC‐labeled heparin (10 U mL^−1^) was added for a 30 min or 12 h incubation, respectively. Cells were fixed with 4% paraformaldehyde for 20 min, permeabilized with 0.3% Triton X‐100 for 1 min, and rinsed. Blocking was done with sheep serum (ZLI‐9056) for 1 h at 37 °C. The primary ZO‐1 antibody (1:200 in 5% BSA) was incubated overnight at 4 °C, followed by washing. Cells were then incubated with fluorescent secondary antibodies for 1 h at 37 °C and washed three times. After staining with DAPI for 5 min and a final wash, cells were examined using a confocal microscope.

### Transmission Electron Microscopy Observation

The superior mesenteric artery vessels of the rats were promptly fixed in arsenate buffer containing 2.5% glutaraldehyde for 24 h (pH 7.4, 4 °C). The tissues underwent three consecutive 10 min washes in 0.13 M phosphate‐buffered saline. Subsequently, the tissues were post‐fixed with 1% osmium tetroxide (OsO_4_) for 2 h at room temperature. The dehydration process involved a sequential series of ethanol concentrations (65%, 70%, 75%, 80%, and 95%) for 10 min each. The tissues were then immersed in tert‐butoxide for 10 min and dried in CO_2_. Staining was performed with uranyl acetate or lead citrate, followed by gold coating using an ion‐sputter coater. The images of the samples were captured and analyzed using a transmission electron microscope (H‐7500; Hitachi, Japan).

### Detection of Mitochondrial Respiratory Function

The superior mesenteric artery vessels mitochondrial respiratory function was evaluated using an Oroboros Oxygraph‐2 k in a thermostat‐controlled chamber (Oroboros Instruments, Innsbruck). In brief, fresh artery vessels (10 mg) were isolated from rat, cut into small pieces, and homogenized with a glass tissue grinder in 400 µL of mitochondrial respiration solution (MiR05) on ice. Then, ≈70 µL of tissue homogenate was used for the analysis of mitochondrial respiratory function. After a 2‐min equilibration period, 20 mM glutamate, 4 mM malate and 1.5 mM ADP were added in sequence to determine the state 4 (leak state) and state 3 (phosphorylation) respiration of complex I (C‐I). Succinate (10 mM) was added to evaluate the state 3 respiration of complex II (C‐II). Then, carbonyl cyanide p‐ trifluoromethoxyphenylhydrazone (FCCP, final concentration: 2–3 µM) was then used to obtain the maximal uncoupled respiratory capacity of the electron transfer system (ETS), and rotenone (1 µM) was used for CII ETS evaluation. Finally, antimycin A was added to measure residual oxygen consumption (ROX). All results as well as oxygen concentrations and oxygen consumption slope (pmol/s/mg) were recorded by using DatLab software 5.2 (Oroboros Instruments).

### FITC–BSA Leakage Detection of Mesenteric Microvessels

The ileocecal part of the mesentery in rats was harvested under surgical anesthesia and then exposed and placed on a transparent glass slide. Throughout the surgical procedure, the mesenteric surface was continuously moistened with physiological saline at 37 °C to maintain warm and moist conditions. The mesenteric microcirculation was observed using a hypersensitive camera (HAMAMATSU, Japan) mounted on a microscope. Following a 10‐min baseline observation, 10 mg kg^−1^ FITC–BSA was intravenously injected. Microvascular leakage was recorded at 6 min post‐injection. The rate of extravasation in the mesenteric microvessels was quantified using Image‐Pro Plus 5.0 software.^[^ ^34^ ^]^

### Mitochondrial Quality Detection

The VECs were cultured in confocal dishes as described above. JC‐1 mitochondrial membrane potential fluorescence probe detection reagent, MitoTracker, or ROS detection solution was added to the cells according to the manufacturer's instructions. After incubating for 30 min in a 37 °C and 5% CO_2_ incubator, cellular imaging was performed using a laser confocal‐scanning microscope (Leica TCS SP5, Germany). For JC‐1 monomer fluorescence and DCFH‐DA detection, excitation was at 488 nm, with emission collected between 501 and 563 nm. MitoTracker was excited at 633 nm, with emission captured between 655 and 670 nm. JC‐1 aggregate fluorescence was excited at 525 nm, with emission detected at 590 nm. Images were quantified using ImageJ software. For ROS detection, VECs were seeded in six‐well plates and treated as previously described. The collected cells were incubated with ROS detection solution at 37 °C in the dark for 30 min. The average fluorescence intensity was then measured using a flow cytometer (Beckman Coulter, USA). To determine the mitochondrial OCR, VECs were seeded in pyruvate solution and glutamine solution on an XFe plate at a density of 1 × 10^4^ cells per well. The plate was left on a clean bench for 1 h to allow the cells to settle naturally. The cells were then returned to the incubator overnight to allow adherence to the walls. Once the cells reached 70%–80% confluence, they were treated with LPS and/or UFH and ENO as previously described. Prior to measurement, the cells were incubated for 50 min in a base medium containing 2.5 µM glucose and 2 mM glutamine, followed by successive incubations with 2 µM oligomycin, 1 µM FCCP, and 0.5 µM rotenone/antimycin A. Under mitochondrial stress conditions, the OCR was determined using an extracellular flux analyzer.

### Mesenteric Blood Flow Measurement

The rats were anesthetized, and mesenteric blood flow was measured using a Doppler imaging device (Perimed, Peri‐Cam PSI‐ZR, Sweden). The mesentery was exposed to a laser beam from a distance of 14 cm. Color images were used to represent the relative perfusion levels at specific sites. The captured images were analyzed using the PIMsoft software (Perimed, Sweden) to accurately assess mesenteric blood flow.

### Transmembrane Electrical Resistance (TEER) Detection

The TEER and BSA leakage of VECs were measured as previously described.^[^ ^35^ ^]^ Briefly, VECs were seeded onto the upper inserts of a Transwell culture plate. After reaching full confluence, they were treated with LPS and/or UFH and ENO as described in an earlier section. The TEER of the VECs was measured every 30 min using a voltohmmeter (World Precision Inc., USA). After completing the TEER analysis, BSA leakage in the VECs was determined. FITC‐labeled BSA (10 µg mL^−1^) was added to the upper inserts of the Transwell, followed by collecting 200 µL of medium from the lower chamber at 10, 20, 30, 40, 50, and 60 min for fluorescence intensity measurements. After collection, an equivalent volume of fresh medium was added to the lower chamber. BSA leakage was calculated using the following formula: BSA leakage (%) = (A10 + A20 + A30 + A40 + A50 + A60)/total fluorescence intensity, where Ax represents the fluorescence intensity at x min.

### Statistical Analysis

Data were presented as mean ± standard deviation unless otherwise stated. A paired t‐test was used to compare two independent groups. To assess the effect size across studies, the SMD was calculated, with an SMD < 0.1 indicating no significant difference in the balance of baseline data between the two groups. Survival analysis was conducted using Kaplan–Meier curves, and the significance of the differences between curves was determined using the log‐rank test. The Chi‐square test was used to compare between categorical variables. Comprehensive univariate and multivariate analyses were performed to identify significant predictors and potential confounding factors. Multivariate Cox proportional hazards models were used to analyze survival data. All statistical analyses were done with SPSS version 20.0 (SPSS Inc., Chicago, IL, USA) and R software version 4.3.2. A *P*‐value less than 0.05 was set to be a statistical significance.

## Conflict of Interest

The authors declare no conflict of interest.

## Author Contributions

R.L., H.H., D.H., and S.H. contributed equally to this article. D.C.Y. and H.H. performed conceptualization. L.R.X. and G.Q. performed methodology. H.D.Y. and H.S. performed investigation. L.H.T., S.R., C.Q. and T.X.X. performed visualization. D.C.Y., M.D.Q., L.L.M.,L.Z.C. and T.Y. performed supervision. D.C.Y., L.R.X., and H.D.Y. wrote the original draft. D.C.Y., L.L.M. and M.D.Q. wrote, reviewed and edited.

## Supporting information



Supporting Information

## Data Availability

The data that support the findings of this study are available from the corresponding author upon reasonable request.

## References

[1] M. Cecconi , L. Evans , M. Levy , A. Rhodes , Lancet 2018, 392, 75. 29937192 10.1016/S0140-6736(18)30696-2

[2] X. Hou , X. Zhang , W. Zhao , C. Zeng , B. Deng , D. W. McComb , S. Du , C. Zhang , W. Li , Y. Dong , Nat. Nanotechnol. 2020, 15, 41. 31907443 10.1038/s41565-019-0600-1 PMC7181370

[3] K. E. Rudd , S. C. Johnson , K. M. Agesa , K. A. Shackelford , D. Tsoi , D. R. Kievlan , D. V. Colombara , K. S. Ikuta , N. Kissoon , S. Finfer , C. Fleischmann‐Struzek , F. R. Machado , K. K. Reinhart , K. Rowan , C. W. Seymour , R. S. Watson , T. E. West , F. Marinho , S. I. Hay , R. Lozano , A. D. Lopez , D. C. Angus , C. J. L. Murray , M. Naghavi , Lancet 2020, 395, 200. 31954465 10.1016/S0140-6736(19)32989-7 PMC6970225

[4] C. Bode , S. Weis , A. Sauer , P. Wendel‐Garcia , S. David , Crit. Care 2023, 27, 478. 38057824 10.1186/s13054-023-04762-6 PMC10698949

[5] V. M. Ranieri , B. T. Thompson , P. S. Barie , J.‐F. Dhainaut , I. S. Douglas , S. Finfer , B. Gårdlund , J. C. Marshall , A. Rhodes , A. Artigas , D. Payen , J. Tenhunen , H. R. Al‐Khalidi , V. Thompson , J. Janes , W. L. Macias , B. Vangerow , M. D. Williams , N. Engl. J. Med. 2012, 366, 2055.22616830

[6] P. R. Mouncey , T. M. Osborn , G. S. Power , D. A. Harrison , M. Z. Sadique , R. D. Grieve , R. Jahan , S. E. Harvey , D. Bell , J. F. Bion , T. J. Coats , M. Singer , J. D. Young , K. M. Rowan , N. Engl. J. Med. 2015, 372, 1301. 25776532 10.1056/NEJMoa1500896

[7] N. I. Shapiro , I. S. Douglas , R. G. Brower , N. Engl. J. Med. 2023, 388, 499.36688507

[8] E. J. Curren , J. D. Lutgring , S. Kabbani , D. J. Diekema , S. Gitterman , E. Lautenbach , D. J. Morgan , C. Rock , R. M. Salerno , L. C. McDonald , Clin. Infect. Dis. 2022, 74, 723. 34346494 10.1093/cid/ciab672

[9] B. Adamik , W. Gozdzik , D. Jakubczyk , M. Welna , A. Kubler , Blood Coagul. Fibrinolysis 2017, 28, 163. 27254441 10.1097/MBC.0000000000000572

[10] D. M. H. Beurskens , J. P. Huckriede , R. Schrijver , H. C. Hemker , C. P. Reutelingsperger , Thromb. Haemost. 2020, 120, 1371. 32820487 10.1055/s-0040-1715460

[11] F. M. Lira Chavez , L. P. Gartzke , F. E. van Beuningen , S. E. Wink , R. H. Henning , G. Krenning , H. R. Bouma , Redox Biol. 2023, 68, 102968. 38039825 10.1016/j.redox.2023.102968 PMC10711241

[12] G. S. Supinski , E. A. Schroder , L. A. Callahan , Chest 2020, 157, 310. 31494084 10.1016/j.chest.2019.08.2182 PMC7005375

[13] S. Hao , H. Huang , R. Y. Ma , X. Zeng , C. Y. Duan , Mil. Med. Res. 2023, 10, 46. 37833768 10.1186/s40779-023-00482-8 PMC10571487

[14] C. Duan , R. Liu , L. Kuang , Z. Zhang , D. Hou , D. Zheng , X. Xiang , H. Huang , L. Liu , T. Li , Adv. Sci. (Weinh) 2023, 10, e2304885. 37909346 10.1002/advs.202304885 PMC10754141

[15] C. Duan , L. Kuang , C. Hong , X. Xiang , J. Liu , Q. Li , X. Peng , Y. Zhou , H. Wang , L. Liu , T. Li , Cell Death Dis. 2021, 12, 1050. 34741026 10.1038/s41419-021-04343-x PMC8571301

[16] X. Zeng , Y. D. Zhang , R. Y. Ma , Y. J. Chen , X. M. Xiang , D. Y. Hou , X.‐H. Li , H. Huang , T. Li , C.‐Y. Duan , Mil. Med. Res. 2022, 9, 25. 35624495 10.1186/s40779-022-00383-2 PMC9137164

[17] Y. Tang , X. Wang , Z. Li , Z. He , X. Yang , X. Cheng , Y. Peng , Q. Xue , Y. Bai , R. Zhang , K. Zhao , F. Liang , X. Xiao , U. Andersson , H. Wang , T. R. Billiar , B. Lu , Immunity 2021, 54, 454. 33561388 10.1016/j.immuni.2021.01.007

[18] L. Evans , A. Rhodes , W. Alhazzani , M. Antonelli , C. M. Coopersmith , C. French , F. R. Machado , L. Mcintyre , M. Ostermann , H. C. Prescott , C. Schorr , S. Simpson , W. J. Wiersinga , F. Alshamsi , D. C. Angus , Y. Arabi , L. Azevedo , R. Beale , G. Beilman , E. Belley‐Cote , L. Burry , M. Cecconi , J. Centofanti , A. Coz Yataco , J. De Waele , R. P. Dellinger , K. Doi , B. Du , E. Estenssoro , R. Ferrer , et al., Crit. Care Med. 2021, 49, e1063. 34605781 10.1097/CCM.0000000000005337

[19] F. Jaimes , G. De La Rosa , C. Morales , F. Fortich , C. Arango , D. Aguirre , Á. Muñoz , Crit. Care Med. 2009, 37, 1185. 19242322 10.1097/CCM.0b013e31819c06bc

[20] J.‐J. Huang , J.‐Z. Cai , Z.‐P. Zhou , Y. Liu , Z.‐J. Yang , D.‐Z. Li , Y.‐H. Chen , Y.‐Y. Luan , Y.‐M. Yao , M. Wu , Front. Pharmacol. 2023, 14, 1281235. 38116082 10.3389/fphar.2023.1281235 PMC10729002

[21] C. Wang , C. Chi , L. Guo , X. Wang , L. Guo , J. Sun , B. Sun , S. Liu , X. Chang , E. Li , Crit. Care 2014, 18, 563. 25318353 10.1186/s13054-014-0563-4 PMC4213495

[22] J.‐C. Peng , F. Nie , Y.‐J. Li , Q.‐Y. Xu , S.‐P. Xing , W. Li , Y. Gao , Front. Med. (Lausanne) 2021, 8, 773339. 35047524 10.3389/fmed.2021.773339 PMC8761617

[23] A. C. Spyropoulos , M. Goldin , D. Giannis , W. Diab , J. Wang , S. Khanijo , A. Mignatti , E. Gianos , M. Cohen , G. Sharifova , J. M. Lund , A. Tafur , P. A. Lewis , K. P. Cohoon , H. Rahman , C. P. Sison , M. L. Lesser , K. Ochani , N. Agrawal , J. Hsia , V. E. Anderson , M. Bonaca , J. L. Halperin , J. I. Weitz , L. Ohanesian , M. Glater , C. Ho , A. Iakovou , D. Ying , M. Dastagir , et al., JAMA Intern. Med. 2021, 181, 1612. 34617959 10.1001/jamainternmed.2021.6203 PMC8498934

[24] C. Wei , W. Jiang , R. Wang , H. Zhong , H. He , X. Gao , S. Zhong , F. Yu , Q. Guo , L. Zhang , L. D. J. Schiffelers , B. Zhou , M. Trepel , F. I. Schmidt , M. Luo , F. Shao , Nature 2024, 629, 893. 38632402 10.1038/s41586-024-07314-2

[25] J. Hou , J. Zhang , P. Cui , Y. Zhou , C. Liu , X. Wu , Y. Ji , S. Wang , B. Cheng , H. Ye , L. Shu , K. Zhang , D. Wang , J. Xu , Q. Shu , M. Colonna , X. Fang , J. Clin. Invest. 2021, 131, e135197. 33586673 10.1172/JCI135197 PMC7880419

[26] H. She , Y. Zhu , H. Deng , L. Kuang , H. Fang , Z. Zhang , C. Duan , J. Ye , J. Zhang , L. Liu , Y. Hu , T. Li , Front. Cell Dev. Biol. 2021, 9, 636327. 33777946 10.3389/fcell.2021.636327 PMC7991806

[27] D. Zheng , J. Zhang , Z. Zhang , L. Kuang , Y. Zhu , Y. Wu , M. Xue , H. Zhao , C. Duan , L. Liu , T. Li , Front. Cell Dev. Biol. 2020, 8, 643. 32766250 10.3389/fcell.2020.00643 PMC7379030

[28] A. L. Goldberger , L. A. N. Amaral , L. Glass , J. M. Hausdorff , P. C. H. Ivanov , R. G. Mark , J. E. Mietus , G. B. Moody , C.‐K. Peng , H. E. Stanley , Circulation 2000, 101, E215. 10851218 10.1161/01.cir.101.23.e215

[29] M. Singer , C. S. Deutschman , C. W. Seymour , M. Shankar‐Hari , D. Annane , M. Bauer , R. Bellomo , G. R. Bernard , J.‐D. Chiche , C. M. Coopersmith , R. S. Hotchkiss , M. M. Levy , J. C. Marshall , G. S. Martin , S. M. Opal , G. D. Rubenfeld , T. van der Poll , J.‐L. Vincent , D. C. Angus , JAMA, J. Am. Med. Assoc. 2016, 315, 801. 10.1001/jama.2016.0287 PMC4968574 26903338

[30] Z.‐S. Zhang , H.‐L. Zhao , G.‐M. Yang , J.‐T. Zang , D.‐Y. Zheng , C.‐Y. Duan , L. Kuang , Y. Zhu , Y. Wu , T. Li , L.‐M. Liu , J. Trauma Acute Care Surg. 2019, 87, 1336. 31389921 10.1097/TA.0000000000002466

[31] V. Martinez‐Sales , V. Vila , E. Reganon , J. G. Oms , J. Aznar , Haematologica 2003, 88, 694. 12801846

[32] J. L. Duffner , P. A. Clemons , A. N. Koehler , Curr. Opin. Chem. Biol. 2007, 11, 74. 17169601 10.1016/j.cbpa.2006.11.031

[33] D. R. Calabrese , K. Zlotkowski , S. Alden , W. M. Hewitt , C. M. Connelly , R. M. Wilson , S. Gaikwad , L. Chen , R. Guha , C. J. Thomas , B. A. Mock , J. S. Schneekloth , Nucleic Acids Res. 2018, 46, 2722. 29481610 10.1093/nar/gky084 PMC5888870

[34] C. Li , Q. Li , Y.‐Y. Liu , M.‐X. Wang , C.‐S. Pan , L. Yan , Y.‐Y. Chen , J.‐Y. Fan , J.‐Y. Han , Am. J. Physiol. Gastrointest. Liver Physiol. 2014, 306, G111. 24232000 10.1152/ajpgi.00123.2013

[35] J. Zhang , G. M. Yang , Y. Zhu , X. Y. Peng , T. Li , L. M. Liu , Am. J. Physiol. Lung Cell Mol. Physiol. 2015, 309, L1323. 26342084 10.1152/ajplung.00016.2015

