# Supplementary material for: Unfractionated Heparin Enhances Sepsis Prognosis Through Inhibiting Drp1‐Mediated Mitochondrial Quality Imbalance
Source: Adv Sci (Weinh). 2024 Oct 24;11(46):2407705. doi: 10.1002/advs.202407705 (PMC11633531; doi:10.1002/advs.202407705)
Supplement: Supplementary file 1 — Supporting Information [file ADVS-11-2407705-s001.docx]

**Supplementary Materials**

**Table S1.**

Baseline characteristics of the study population with and without exposure to UFH.

| **Characteristic** | **Pre-matched patients** | | **SMD*** | **Post-matched patients** | | **SMD** |
| --- | --- | --- | --- | --- | --- | --- |
|  | **Control** | **UFH** |  | **Control** | **UFH** |  |
|  | (n=4597) | (n=1928) |  | (n=1924) | (n=1924) |  |
| **Demographics** |  |  |  |  |  |  |
| Age (years), mean ± SD | 64.14 ± 17.67 | 63.53 ± 17.13 | 0.035 | 63.75 ± 17.56 | 63.54 ± 17.14 | 0.012 |
| Gender (male), n, (%) | 2667 (58.0%) | 1112 (57.7%) | 0.009 | 1128 (58.6%) | 1110 (57.7%) | 0.019 |
| **Clinical vital signs** |  |  |  |  |  |  |
| Heart rate, mean ± SD | 86.79 ± 16.11 | 87.64 ± 16.88 | 0.052 | 87.24 ± 15.96 | 87.61 ± 16.86 | 0.022 |
| MBP, mean ± SD | 78.19 ± 9.96 | 78.15 ± 10.31 | 0.004 | 78.19 ± 9.97 | 78.14 ± 10.31 | 0.005 |
| SpO_2,_ mean ± SD | 97.23 ± 2.04 | 97.10 ± 2.10 | 0.065 | 97.08 ± 2.08 | 97.10 ± 2.10 | 0.01 |
| **Scoring systems** |  |  |  |  |  |  |
| SAPS II, mean ± SD | 37.46 ± 12.98 | 38.36 ± 13.36 | 0.069 | 38.33 ± 13.47 | 38.34 ± 13.35 | <0.001 |
| OASIS, mean ± SD | 35.64 ± 8.72 | 36.26 ± 8.57 | 0.072 | 36.27 ± 9.01 | 36.25 ± 8.55 | 0.002 |
| SOFA, mean ± SD | 3.33 ± 1.69 | 3.37 ± 1.80 | 0.021 | 3.31 ± 1.62 | 3.35 ± 1.74 | 0.024 |
| **Comorbidities** |  |  |  |  |  |  |
| Myocardial infarction, n (%) | 658 (14.3%) | 348 (18.1%) | 0.101 | 342 (17.8%) | 345 (17.9%) | 0.004 |
| Heart failure, n (%) | 930 (20.3%) | 479 (24.9%) | 0.110 | 470 (24.4%) | 477 (24.8%) | 0.008 |
| Diabetes, n (%) | 970 (21.1%) | 470 (24.4%) | 0.077 | 446 (23.2%) | 467 (24.3%) | 0.026 |
| Dementia, n (%) | 206 ( 4.5%) | 85 ( 4.4%) | 0.004 | 104 ( 5.4%) | 85 ( 4.4%) | 0.046 |
| Cerebrovascular disease, n (%) | 764 (16.7%) | 295 (15.3%) | 0.037 | 303 (15.7%) | 295 (15.3%) | 0.011 |
| Chronic pulmonary disease, n (%) | 1102 (24.0%) | 506 (26.3%) | 0.052 | 525 (27.3%) | 505 (26.2%) | 0.023 |
| **Serum biomarkers** |  |  |  |  |  |  |
| Hemoglobin, mean ± SD | 11.94 ± 2.13 | 11.97 ± 2.18 | 0.017 | 12.01 ± 2.18 | 11.97 ± 2.18 | 0.016 |
| Platelets, mean ± SD | 183.0 ± 100.4 | 193.6 ± 104.1 | 0.104 | 195.6 ± 111.1 | 193.4 ± 103.9 | 0.021 |
| WBC, mean ± SD | 15.31 ± 9.15 | 15.52 ± 8.03 | 0.024 | 15.48 ± 8.76 | 15.51 ± 8.03 | 0.004 |

**Abbreviations:** UFH, unfractionated heparin; SD, standard deviation; MBP, mean blood pressure; SAPS II, Simplified Acute Physiology score II; OASIS, Oxford Acute Severity of Illness Score; SOFA, Sequential Organ Failure Assessment; WBC, white blood cell; SMD, standardized mean difference.

*SMD < 0.1 indicates no significant difference in the balance of baseline data between the two groups.

**Table S2.**

Baseline characteristics of the study population with and without exposure to ENO.

| **Characteristic** | **Pre-matched patients** | | **SMD*** | **Post-matched patients** | | **SMD** |
| --- | --- | --- | --- | --- | --- | --- |
|  | **Control** | **ENO** |  | **Control** | **ENO** |  |
|  | (n=4129) | (n=129) |  | (n=129) | (n=129) |  |
| **Demographics** |  |  |  |  |  |  |
| Age (years), mean ± SD | 66.28 ± 15.83 | 63.51 ± 16.55 | 0.171 | 65.00 ± 17.19 | 63.51 ± 16.55 | 0.088 |
| Gender (male), n (%) | 1849 (44.8%) | 62 (48.1%) | 0.066 | 59 (45.7%) | 62 (48.1%) | 0.047 |
| **Clinical vital signs** |  |  |  |  |  |  |
| Heart rate, mean ± SD | 87.93 ± 16.19 | 96.39 ± 17.32 | 0.505 | 95.51 ± 16.94 | 96.39 ± 17.32 | 0.051 |
| MBP, mean ± SD | 76.63 ± 9.64 | 77.66 ± 11.49 | 0.098 | 77.13 ± 10.12 | 77.66 ± 11.49 | 0.05 |
| SpO_2_, mean ± SD | 97.19 ± 2.11 | 96.82 ± 1.86 | 0.188 | 96.87 ± 2.15 | 96.82 ± 1.86 | 0.027 |
| **Scoring systems** |  |  |  |  |  |  |
| SAPS II, mean ± SD | 39.03 ± 12.72 | 39.61 ± 14.55 | 0.042 | 40.42 ± 13.27 | 39.61 ± 14.55 | 0.058 |
| OASIS, mean ± SD | 35.30 ± 8.56) | 36.15 ± 8.58 | 0.099 | 36.66 ± 8.80) | 36.15 ± 8.58 | 0.059 |
| SOFA, mean ± SD | 3.47 ± 1.83 | 3.12 ± 1.34 | 0.221 | 3.13 ± 1.41) | 3.12 ± 1.34 | 0.011 |
| **Comorbidities** |  |  |  |  |  |  |
| Heart failure, n (%) | 1167 (28.3%) | 25 (19.4%) | 0.210 | 30 (23.3%) | 25 (19.4%) | 0.095 |
| Diabetes, n (%) | 982 (23.8%) | 23 (17.8%) | 0.147 | 22 (17.1%) | 23 (17.8%) | 0.020 |
| Cerebrovascular disease, n (%) | 629 (15.2) | 6 ( 4.7) | 0.359 | 3 ( 2.3) | 6 ( 4.7) | 0.127 |
| Chronic pulmonary disease, n (%) | 1129 (27.3) | 34 (26.4) | 0.022 | 33 (25.6) | 34 (26.4) | 0.018 |
| **Serum biomarkers** |  |  |  |  |  |  |
| Hemoglobin, mean ± SD | 11.40 ± 2.04 | 11.01 ± 2.36 | 0.180 | 11.16 ± 2.08 | 11.01 ± 2.36 | 0.069 |
| Platelets, mean ± SD | 180.09 ± 112.76 | 197.08 ± 118.98 | 0.147 | 199.59 ± 123.12 | 197.08 ± 118.98 | 0.021 |
| WBC, mean ± SD | 15.37 ± 11.44 | 13.73 ± 9.37 | 0.157 | 13.15 ± 7.23 | 13.73 ± 9.37 | 0.069 |

**Abbreviations:** ENO, enoxaparin; SD, standard deviation; MBP, mean blood pressure; SAPS II, Simplified Acute Physiology Score II; OASIS, Oxford Acute Severity of Illness Score; SOFA, Sequential Organ Failure Assessment; WBC, white blood cell; SMD, standardized mean difference.

*SMD < 0.1 indicates no significant difference in the balance of baseline data between the two groups.


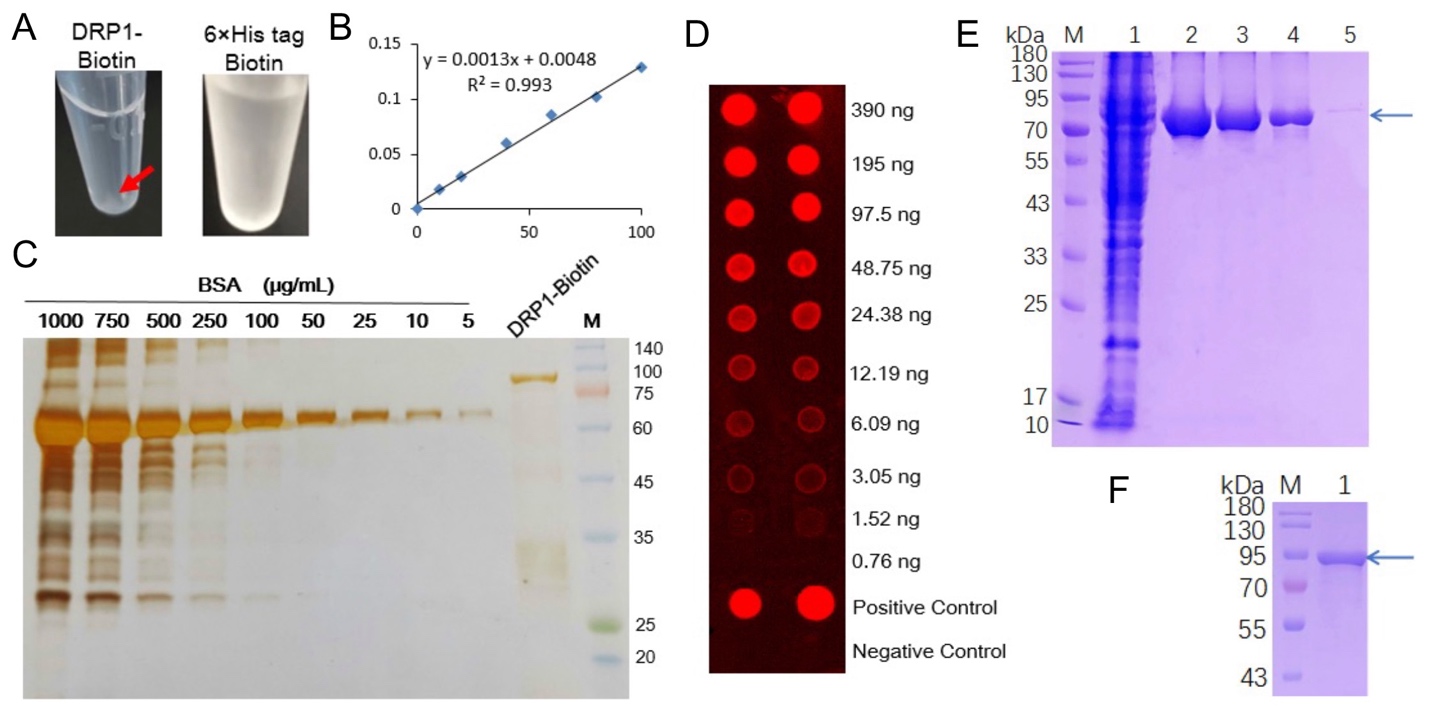


**Figure S1. Comprehensive Protein Analysis and Purification Techniques for Drp1.**

(A) Comparative centrifugation outcomes for DRP1-Biotin (experimental sample) and 6xHis tag Biotin (control sample). (B) Implementation of the Bradford assay for protein concentration analysis in samples. (C) Utilization of SDS-PAGE to evaluate protein sample purity. (D) Dot Blot assay results, featuring BSA-biotin as the positive control and BSA as the negative control. (E) SDS-PAGE display of Drp1 protein purification process (M: Marker; Lane 1: Flow-through; Lanes 2-5: Purified samples). (F) Final SDS-PAGE results showcasing the Drp1 protein (M: Marker; Lane 1: Drp1 protein).


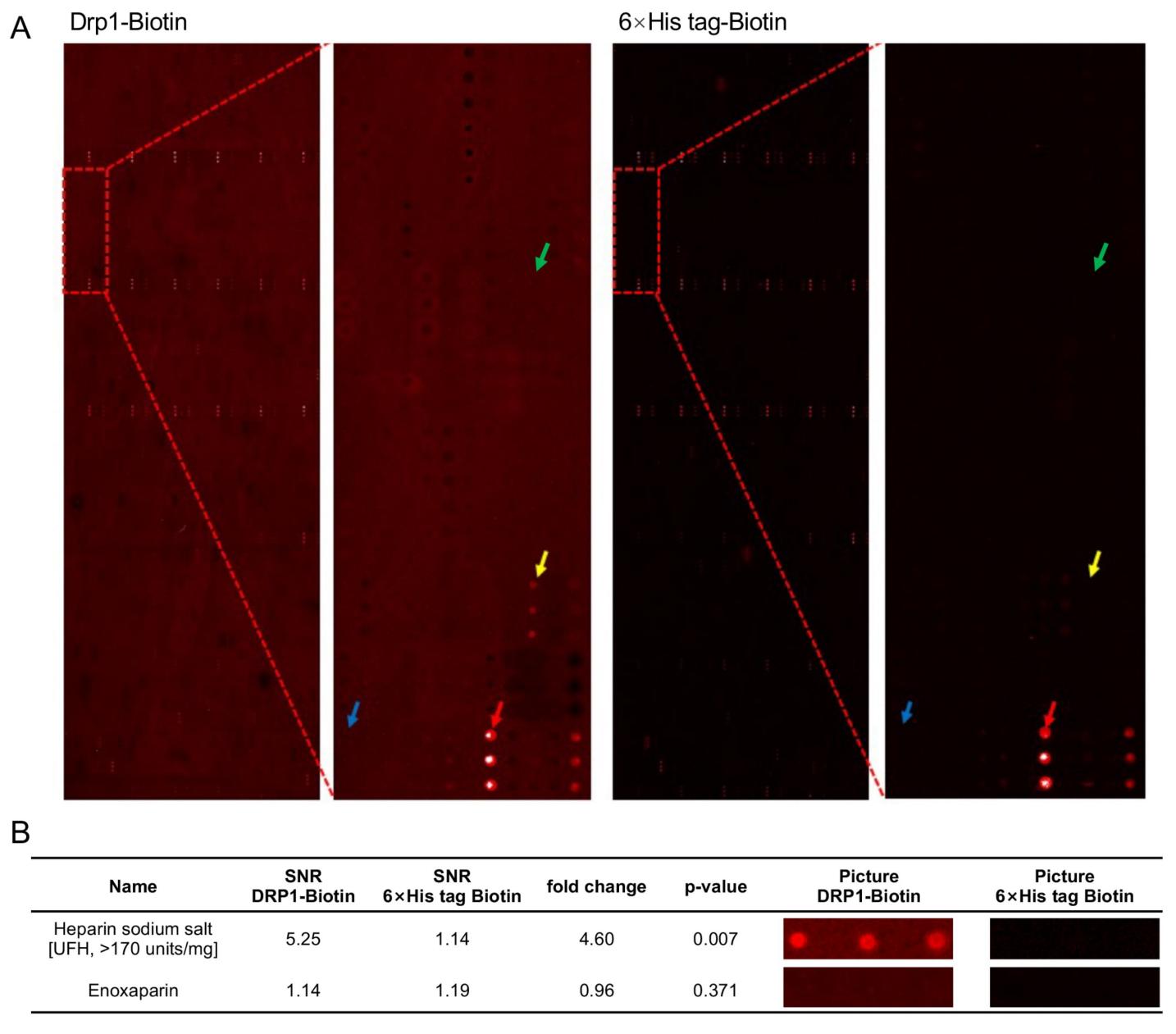


**Figure S2. Microarray Chip Scanning Outcomes**

(A) This figure presents comprehensive scan images of the samples, along with magnified views of specific chip sections. Red arrows highlight the positive control (biotin), blue arrows indicate the negative control (DMSO), yellow arrows signify positive small-molecule hits (Heprin) and green arrows positive indicate small-molecule hits (ENO). (B) Potentially positive small-molecule analysis results.


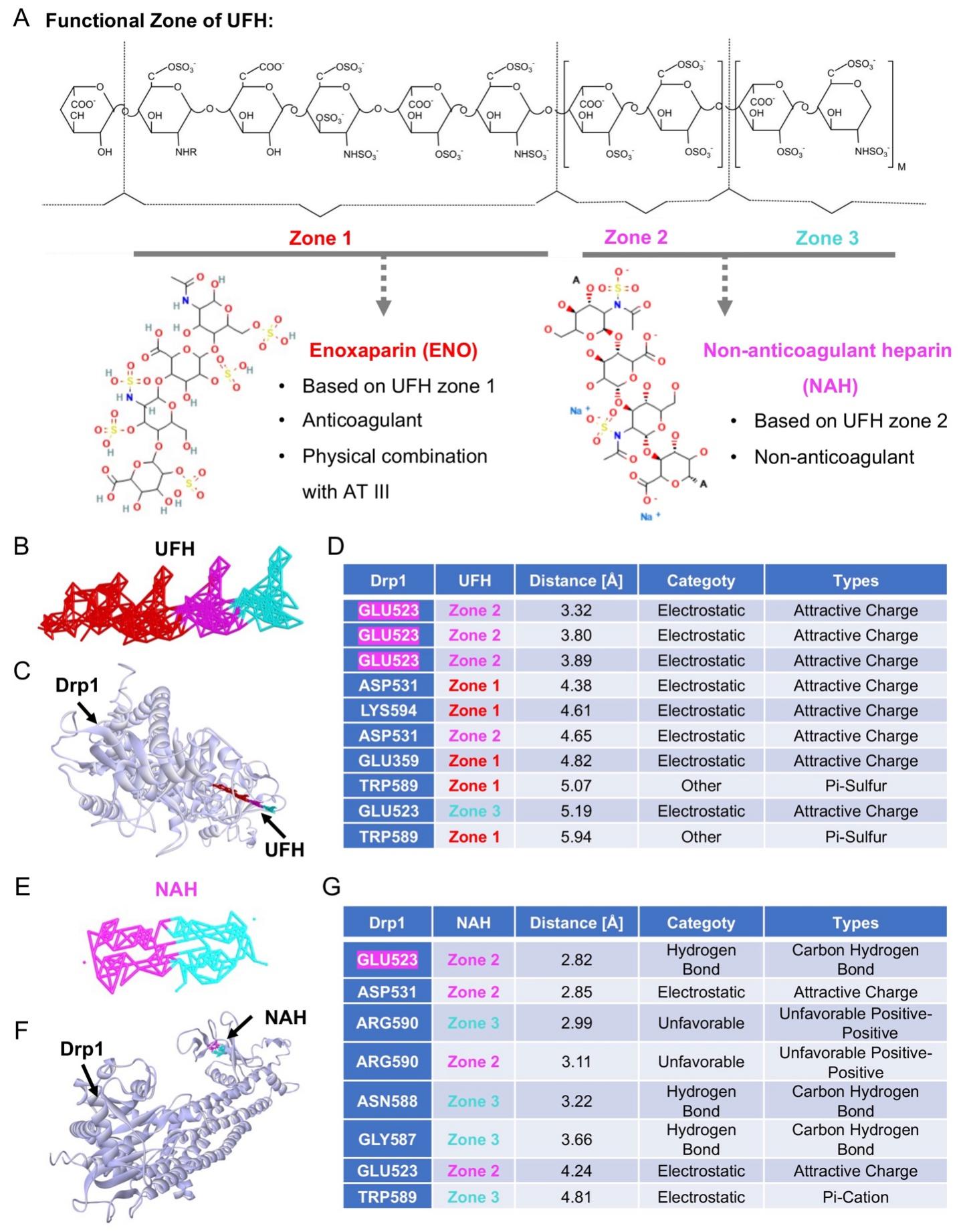


**Figure S3. UFH Functional Zones and Molecular Interactions**

1. Structural Formula and Functional Zones of UFH: This segment details the specific zones of Unfractionated Heparin (UFH), with zone 1 underpinning enoxaparin (ENO) and zones 2 and 3 aligned with non-anticoagulant heparin (NAH). (B) Three-dimensional structural representation of UFH. (C) Molecular docking of Drp1 to UFH. (D) Interaction forces between Drp1 and UFH. (E) Three-dimensional ENO structure. (F) Molecular docking of Drp1 to ENO. (G) Types of interaction force between Drp1 and ENO.


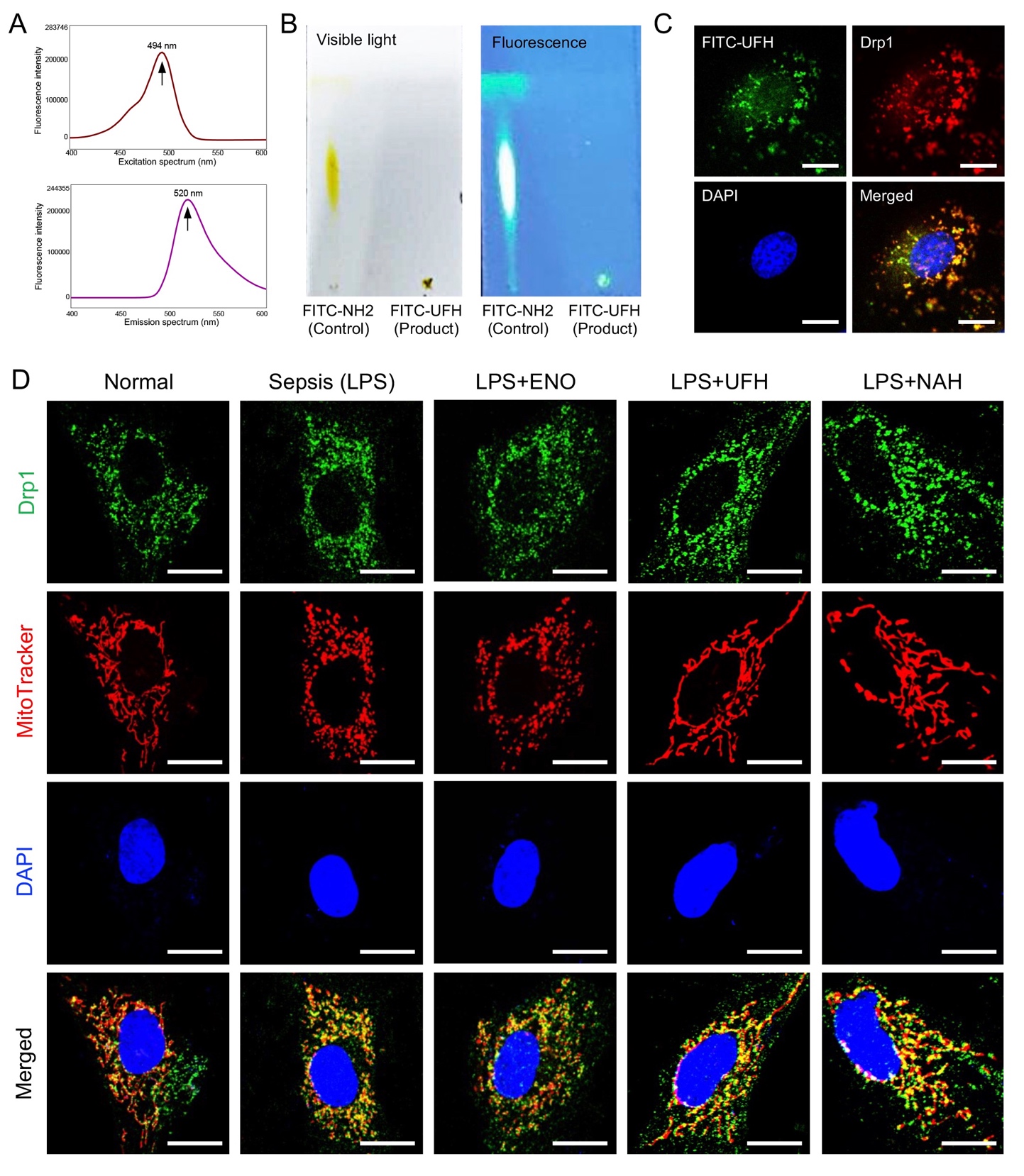


**Figure S4. Characterization of FITC Labeling and Drp1 Localization**

1. The excitation and emission spectra of FITC show peaks at 494 nm (excitation) and 520 nm (emission). (B) Thin-layer chromatography under visible light and fluorescence for the control (FITC-NH2) and product (FITC-UFH). (C) Confocal images of FITC-UFH co-localized with Drp1 in cells (Bar, 20 µm), n = 3. (D) Drp1 expression in the subcellular regions of the VECs in each group. a: *P* < 0.05, compared with the normal group; b: *P* < 0.05, compared with the LPS group.


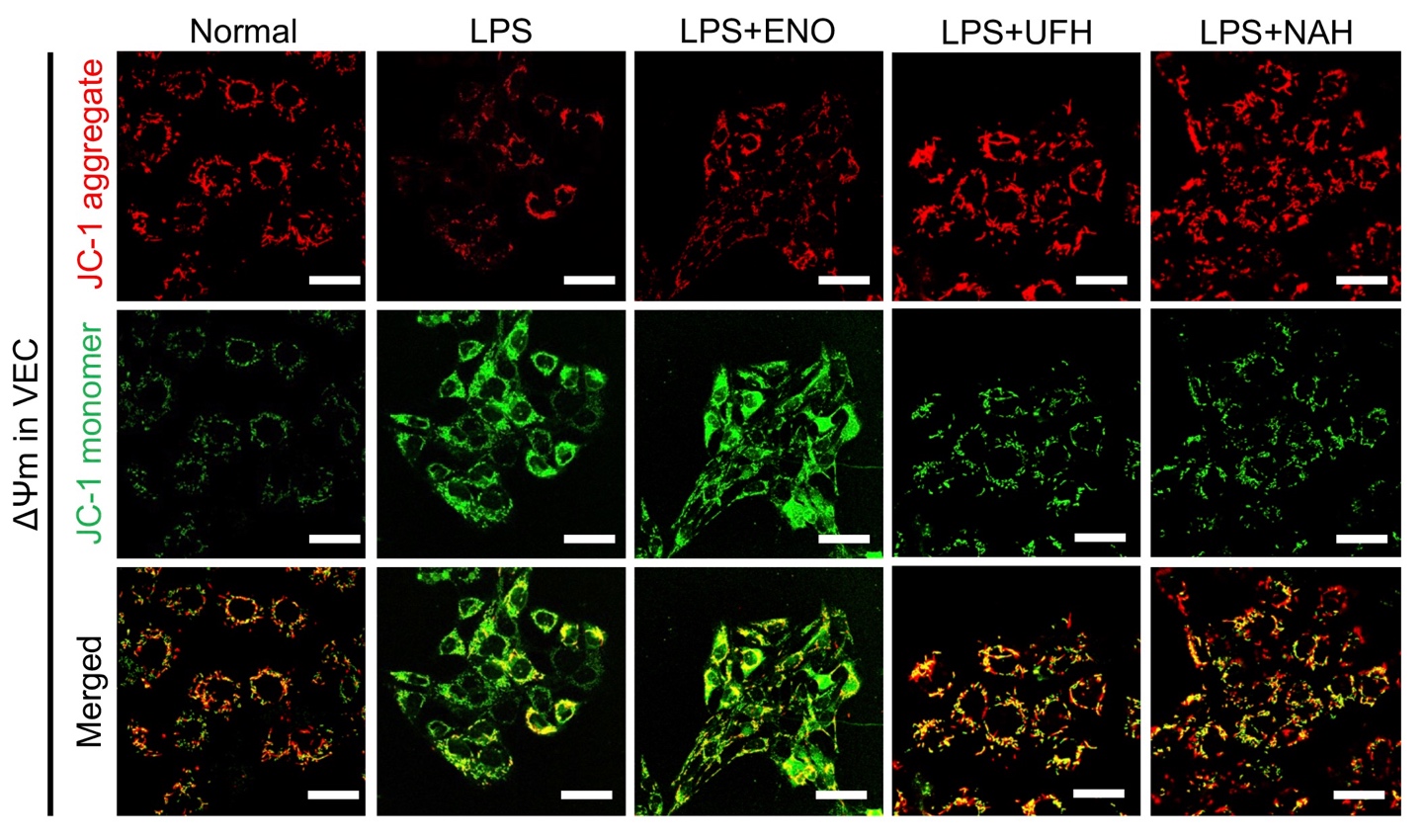


**Figure S5. Comparative Effects of ENO, UFH, and NAH Treatments on mitochondrial membrane potential (ΔΨm) in Sepsis**

Red fluorescence indicated JC-1 aggregates, signifying polarized mitochondria, whereas green fluorescence indicated JC-1 monomers, indicating depolarized mitochondria.


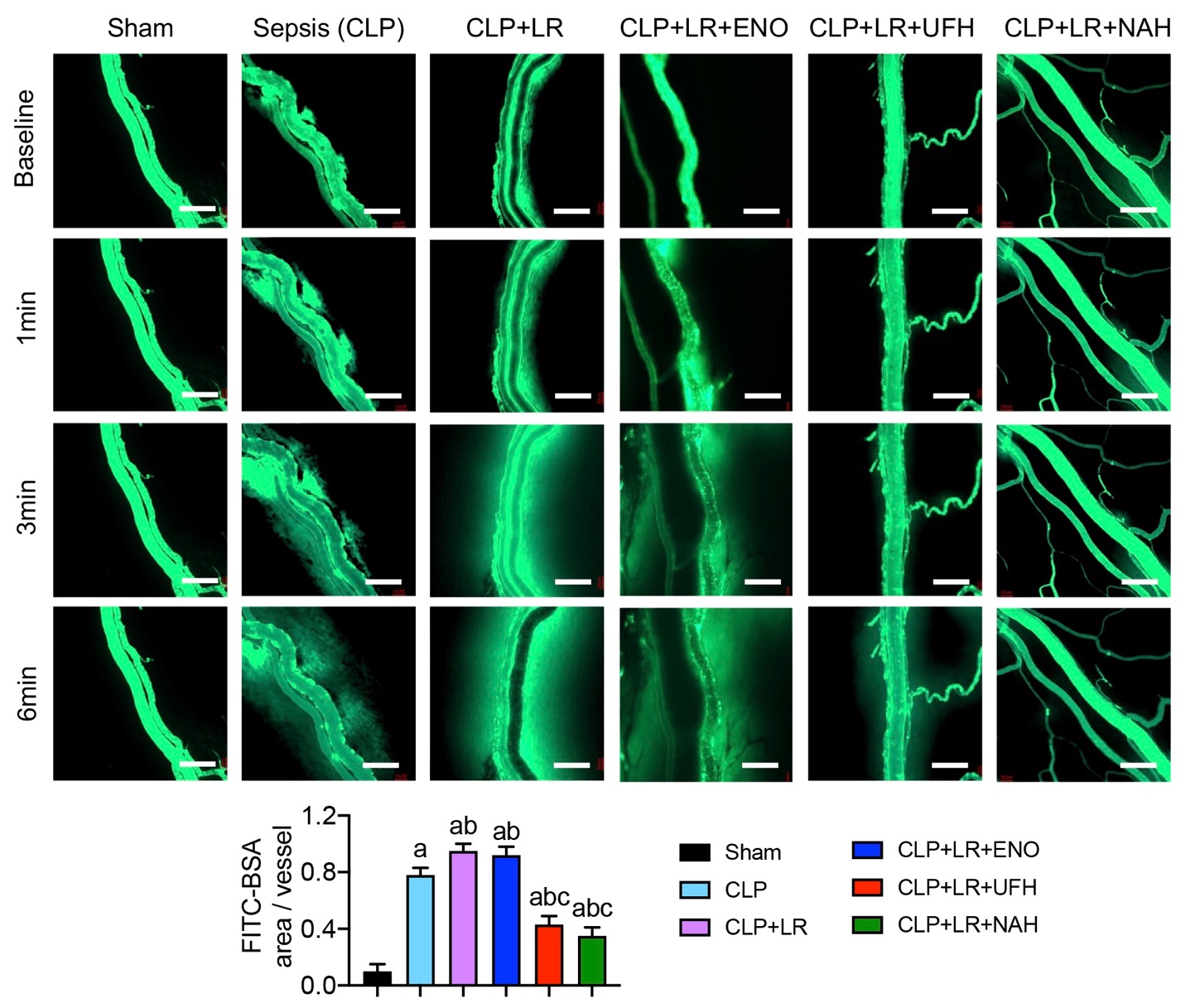


**Figure S6. Time-course Analysis of FITC-BSA Permeability in Mesenteric Microvessels**

Sequential confocal images displayed the permeation of FITC-BSA at baseline and 1, 3, and 6 min post-intervention in each group. a: *P* < 0.05 compared with the sham group; b: *P* < 0.05 compared with the CLP group; c: *P* ＜ 0.05 compared with the LR group.
